# Does type of funding affect reporting in network meta-analysis? A scoping review of network meta-analyses

**DOI:** 10.1186/s13643-023-02235-z

**Published:** 2023-05-06

**Authors:** Areti Angeliki Veroniki, Eric Kai Chung Wong, Carole Lunny, Juan Camilo Martinez Molina, Ivan D. Florez, Andrea C. Tricco, Sharon E. Straus

**Affiliations:** 1 Knowledge Translation Program, Li Ka Shing Knowledge Institute, St. Michael’s Hospital, Toronto, ON Canada; 2 Institute for Health Policy, Management, and Evaluation, University of Toronto, Toronto, ON Canada; 3 Medical Research Institute, School of Medicine, University of Antioquia, Medellín, Colombia; 4 Cochrane Hypertension Review Group and the Therapeutics Initiative, University of British Columbia, Vancouver, Canada; 5 Paediatric Intensive Care Unit, Clinica Las Américas, Medellin, Colombia; 6 Department of Pediatrics, University of Antioquia, Medellín, Colombia; 7 Department of Health Research Methods, Evidence and Impact, McMaster University, Hamilton, Canada; 8 Epidemiology Division & Institute of Health Policy, Management, and Evaluation, Dalla Lana School of Public Health, University of Toronto, Toronto, ON Canada; 9 Department of Geriatric Medicine, University of Toronto, Toronto, ON Canada

**Keywords:** Sponsorship, Funding bias, Industry-funding, Network meta-analysis, Multiple treatment meta-analysis

## Abstract

**Background:**

Evidence has shown that private industry-sponsored randomized controlled trials (RCTs) and meta-analyses are more likely to report intervention-favourable results compared with other sources of funding. However, this has not been assessed in network meta-analyses (NMAs).

**Objectives:**

To (a) explore the recommendation rate of industry-sponsored NMAs on their company’s intervention, and (b) assess reporting in NMAs of pharmacologic interventions according to their funding type.

**Methods:**

Design: Scoping review of published NMAs with RCTs.

Information Sources: We used a pre-existing NMA database including 1,144 articles from MEDLINE, EMBASE and Cochrane Database of Systematic Reviews, published between January 2013 and July 2018.

Study Selection: NMAs with transparent funding information and comparing pharmacologic interventions with/without placebo.

Synthesis: We captured whether NMAs recommended their own or another company’s intervention, classified NMAs according to their primary outcome findings (i.e., statistical significance and direction of effect), and according to the overall reported conclusion. We assessed reporting using the Preferred Reporting Items for Systematic Reviews and Meta-Analysis extension to NMA (PRISMA-NMA) 32-item checklist. We matched and compared industry with non-industry NMAs having the same research question, disease, primary outcome, and pharmacologic intervention against placebo/control.

**Results:**

We retrieved 658 NMAs, which reported a median of 23 items in the PRISMA-NMA checklist (interquartile range [IQR]: 21–26). NMAs were categorized as 314 publicly-sponsored (PRISMA-NMA median 24.5, IQR 22–27), 208 non-sponsored (PRISMA-NMA median 23, IQR 20–25), and 136 industry/mixed-sponsored NMAs (PRISMA-NMA median 21, IQR 19–24). Most industry-sponsored NMAs recommended their own manufactured drug (92%), suggested a statistically significant positive treatment-effect for their drug (82%), and reported an overall positive conclusion (92%). Our matched NMAs (25 industry vs 25 non-industry) indicated that industry-sponsored NMAs had favourable conclusions more often (100% vs 80%) and were associated with larger (but not statistically significantly different) efficacy effect sizes (in 61% of NMAs) compared with non–industry-sponsored NMAs.

**Conclusions:**

Differences in completeness of reporting and author characteristics were apparent among NMAs with different types of funding. Publicly-sponsored NMAs had the best reporting and published their findings in higher impact-factor journals. Knowledge users should be mindful of this potential funding bias in NMAs.

**Supplementary Information:**

The online version contains supplementary material available at 10.1186/s13643-023-02235-z.

## Background

Industry-sponsored randomised clinical trials (RCTs) are often conducted for regulatory purposes [1]. Results that are unfavourable to a study’s funder can pose considerable financial risks to companies. The cost to develop a new intervention, including expenditures on drugs that fail to reach the market, can range between $1–2 billion USD dollars [2]. Consequently, empirical evidence has shown that private industry-sponsored RCTs tend to report more favourable efficacy (or safety) results and conclusions for the sponsoring company’s intervention compared to RCTs with non-conflicted sources of funding [3, 4]. Several efforts in reducing industry-sponsorship bias have been made, including RCT registration, publication of RCT results in registries, and mandatory disclosure of the sponsor’s role in journal publications, among others [5, 6]. Also, reporting of RCT sponsorship and authors’ conflicts of interests (COIs) in meta-analyses has improved over years [7].

Pressure to make marginal products look novel, more effective and safer than they are, may result in biases in industry-sponsored studies. Sponsorship bias (or funding bias) refers to the tendency of a scientific study to support the interests of the sponsoring company's interventions. [8, 9]. Previous research revealed the impact of sponsorship bias in RCTs [10] and systematic reviews with meta-analysis [11–14]. Empirical evidence showed that private industry-sponsored RCTs reported favourable efficacy results and conclusions, and fewer harms for the sponsoring company’s intervention compared to RCTs with non-conflicted sources of funding [3, 4, 15, 16]. Industry-sponsored systematic reviews with meta-analysis are often produced by company employees, consultancies/contactors hired by the companies, or by authors with industry ties [17], and the findings aligned with sponsor interests [14, 18–21].

Although sponsorship bias may exist in systematic reviews with network meta-analysis (NMA) [8, 22, 23], to date, no study has evaluated it at the NMA level. NMAs are very attractive for decision making and they are increasingly used when developing clinical practice guidelines and health technology assessments [24, 25]. This is because NMAs can compare the efficacy and safety of all interventions in a single model and can rank the interventions according the specific condition from best to worst [26]. NMAs can help decision makers choose among competing interventions, and hence have a policy impact. This heightens the concerns about conflicts of interest. However, the completeness of reporting in NMA is low with a small yearly improvement [27]. It is unclear what the impact of funding source is on the reporting of NMA. To determine whether the results and conclusions of NMAs are trustworthy, we need to examine whether NMAs supported by private manufacturers compared to not-for-profit and no funded NMAs lead to more favourable findings.

In this study, we aimed to (a) explore the recommendation rate of private industry-sponsored network meta-analyses on their own company’s experimental intervention, and (b) assess reporting in NMAs of pharmacologic interventions according to their funding type (industry, public, or non-sponsored).

## Methods

### Study design

This is a scoping review, which aims to evaluate research practices and is based on the JBI (formerly Joanna Briggs Institute) methods manual [28], and followed the Preferred Items for Systematic Reviews and Meta-analysis (PRISMA) extension for scoping reviews [29] (Additional file 2). Our methods are briefly described below, and are detailed in Appendix 1 (Additional file 1) and our previous publications [27, 30–32].

We used our previous collection of NMAs with RCTs published between January 2013 and July 2018 [27]. We included networks of RCTs with at least four intervention nodes and a number of studies larger than the number of nodes compared (e.g., if four nodes were compared then five studies had to be included), where the authors had conducted an adjusted indirect comparison or NMA [33]. These decisions were initially made to ensure sufficient data were available per NMA for the evaluation of treatment efficacy or safety, and to assess characteristics in networks with complex evidence structure that were not captured in previous studies [34, 35]. We developed a predefined data abstraction form in an Excel spreadsheet (see data abstraction form in Appendix 1 (Additional file 1)) [27], and pilot tested it using five purposefully chosen NMAs. One investigator extracted data and another verified the data for accuracy.

We assessed the reporting completeness of the NMAs using the Preferred Reporting Items for Systematic Reviews and Meta-Analysis extension to NMA (PRISMA-NMA) [36] by funding type. The PRISMA statement was first published in 2009 [37] and updated in 2020 [38] to incorporate systematic review methods advances, and was developed to promote transparent reporting in published systematic reviews. The PRISMA extension for NMA was published in 2015 to capture reporting in methods developed specifically for NMA [36].

### Statistical analysis

We performed a descriptive analysis by NMA funding type, and used frequencies and percentages for discrete variables, and median and interquartile range (IQR) for continuous variables. We visually assessed whether NMA publication according to funding type changed over time using a stacked bar plot. For our analysis, we combined industry- with mixed-sponsored NMAs (i.e., funded by both public organizations and industry) in a single group (henceforth called industry-sponsored, as for each NMA, some industry sponsorship was provided).

We explored whether the funding type influenced reporting and journal publication by comparing the underlying distributions of the PRISMA-NMA score and journal impact factor. We presented forest plots of pharmacologic intervention effects and their 95% confidence intervals (CIs) for the underlying industry-sponsored pharmacologic interventions according to the effect size used (e.g., log odds ratio). We compared the distributions of the PRISMA-NMA score by the authors’ conclusion about the primary pharmacologic intervention’s outcome according to the calculated effect size (i.e., (non-)significantly positive/negative/neutral) and by overall conclusion across all outcomes (positive, negative, neutral, indeterminate [Appendix 1 (Additional file 1)] [39]). We also assessed reporting of COIs and their types for the first and senior author in the NMA using barplots (see Appendix 1 (Additional file 1) types of COIs).

We matched industry/mixed- with publicly sponsored NMAs/ NMAs with no funding having: the same research question, disease, primary outcome, and pharmacologic intervention compared with placebo/control. We compared the effect sizes of the matched industry- and non-industry (publicly or not funded) sponsored NMAs using forest plots and by obtaining the differences in log odds ratios and mean differences of the sponsored intervention between the matched NMAs.

### Patients and public involvement

No patients or public were involved in the study.

## Results

Of the total 1,144 NMAs published between 2013 and 2018, we screened 907 NMAs for relevant funding information after removing 237 NMAs with non-pharmacological interventions. Of the screened NMAs, 248 did not report funding information, though in 55 cases this was identified from the PROSPERO record (International Prospective Register of Systematic Reviews). We contacted the remaining 192 NMA corresponding authors, of whom 55 authors responded to our emails; we were unable to locate authors’ contact information for 33 NMAs. We then screened the 769 NMAs for eligibility according to our inclusion criteria and included 658 NMAs categorized as: 131 industry-sponsored, 5 mixed-sponsored (i.e., resulting in a total of 136 NMAs including at least one industry sponsor), 314 publicly-sponsored, and 208 non-sponsored NMAs (i.e., with no funding for the review; Appendix 2 (Additional file 1)).

### Characteristics of the 658 NMAs

According to the country of corresponding author, the majority of NMAs (198 [30%]) were conducted in China, followed by the United States (113 [17%]) and the United Kingdom (106 [16%]; Appendix 3 (Additional file 1)). Most publicly- (161 [51%]) and non-sponsored (99 [44%]) NMAs were conducted in Asia, while most industry-sponsored NMAs were conducted in Europe (71 [52%]) followed by North America (52 [38%]; Table 1). Oncology was the most prevalent condition explored in NMAs (99 [15%]), especially in publicly- (50 [16%]) and non-sponsored (32 [15%]) NMAs, while rheumatology was the most frequent condition explored in industry-sponsored (21 [15%]) NMAs. Network characteristics, including number of treatments, studies, and participants, did not vary between the different funding types, on average. The number of pharmacologic interventions manufactured by the funder and assessed in the industry-sponsored NMAs ranged between zero and five.

**Table 1:** Characteristics of included network meta-analyses, categorized by sponsorship status

	**Industry-sponsored** **(** ***N*** ** = 136)**	**Non-sponsored** **(** ***N*** ** = 208)**	**Publicly-sponsored** **(** ***N*** ** = 314)**	**Overall** **(** ***N*** ** = 658)**
**Continent of corresponding author**				
Africa	1 (1%)	3 (1%)	0 (0%)	4 (1%)
Asia	11 (8%)	92 (45%)	161 (50%)	264 (40%)
Australia	1 (1%)	3 (1%)	2 (1%)	6 (1%)
Europe	71 (52%)	56 (27%)	80 (26%)	207 (31%)
North America	52 (38%)	44 (21%)	63 (20%)	159 (24%)
Oceania	0 (0%)	3 (1%)	2 (1%)	5 (1%)
South America	0 (0%)	7 (4%)	6 (2%)	13 (2%)
**Disease category**				
Allergy and immunology	2 (2%)	0 (0%)	0 (0%)	2 (0%)
Cardiology	9 (7%)	27 (13%)	20 (5%)	56 (9%)
Dermatology	6 (4%)	8 (4%)	9 (3%)	23 (3%)
Endocrinology	19 (14%)	15 (7%)	37 (11%)	71 (11%)
Gastroenterology	6 (4%)	23 (11%)	27 (8%)	56 (9%)
Hematology	7 (5%)	6 (3%)	8 (3%)	21 (3%)
infectious disease	9 (7%)	17 (8%)	24 (8%)	50 (8%)
Neurology	12 (9%)	14 (7%)	16 (5%)	42 (6%)
Obstetrics and gynecology	1 (1%)	4 (2%)	6 (2%)	11 (2%)
Oncology	17 (12%)	32 (15%)	50 (16%)	99 (15%)
Ophthalmology	1 (1%)	1 (1%)	6 (2%)	8 (1%)
Psychiatry	7 (5%)	14 (7%)	24 (8%)	45 (7%)
Respirology	15 (11%)	10 (5%)	15 (5%)	40 (6%)
Rheumatology	21 (15%)	13 (6%)	40 (13%)	74 (11%)
Urology	4 (3%)	2 (1%)	7 (2%)	13 (2%)
Dentistry	0 (0%)	4 (2%)	2 (1%)	6 (1%)
General Surgery	0 (0%)	3 (1%)	4 (1%)	7 (1%)
Nephrology	0 (0%)	10 (5%)	14 (5%)	24 (3%)
Orthopedic surgery	0 (0%)	3 (1%)	3 (1%)	6 (1%)
Pediatrics	0 (0%)	2 (1%)	2 (1%)	4 (1%)
**Outcome type**^**a**^				
Objective	48 (35%)	41 (20%)	77 (25%)	166 (25%)
Semi-objective	25 (19%)	48 (23%)	72 (23%)	145 (22%)
Subjective	63 (46%)	119 (57%)	165 (52%)	347 (53%)
**Efficacy or safety outcome**^**a**^				
Efficacy	131 (96%)	179 (86%)	288 (92%)	598 (91%)
Safety	5 (4%)	29 (14%)	26 (8%)	60 (9%)
**Type of treatment comparison**				
Pharmacologic vs pharmacologic	37 (27%)	71 (34%)	93 (30%)	201 (30%)
Pharmacologic vs placebo	99 (73%)	137 (66%)	221 (70%)	457 (70%)
**Number of RCTs in NMA**^**a**^				
Median [IQR]	20 [12–33]	17 [11–33]	22 [13–44]	20 [12–37]
Not reported	4 (3%)	3 (1%)	0 (0%)	7 (1%)
**Number of participants in NMA**^**a**^				
Median [IQR]	7765 [4332–17150]	4992 [2987–13806]	6515 [2722–18247]	6283 [3071–16230]
Not reported	32 (24%)	24 (11%)	16 (5%)	72 (11%)
**Number of interventions in NMA**^**a**^				
Median [IQR]	8 [6–11]	7 [5–9]	7 [5–10]	7 [5–10]
Not reported	4 (3%)	2 (1%)	0 (0%)	6 (1%)
**Number of nodes in NMA**^**a**^				
Median [IQR]	8 [6–11]	7 [5–10]	8 [6–12]	8 [5–11]
Not reported	6 (4%)	3 (1%)	0 (0%)	9 (1%)
**Number of interventions manufactured by the industry sponsor in NMA**^**a**^				
Median [IQR]	1 [1, 2]	NA [NA–NA]	NA [NA–NA]	1 [1, 2]
Not reported	0 (0%)	208 (100%)	314 (100%)	522 (79%)

*Abbreviations*: *IQR* interquartile range, *NA* not applicable, *NMA* network meta-analysis, *RCT* randomised controlled trial

^**a**^Characteristics as reported in the primary outcome of the systematic review with NMA

**Fig. 1:**
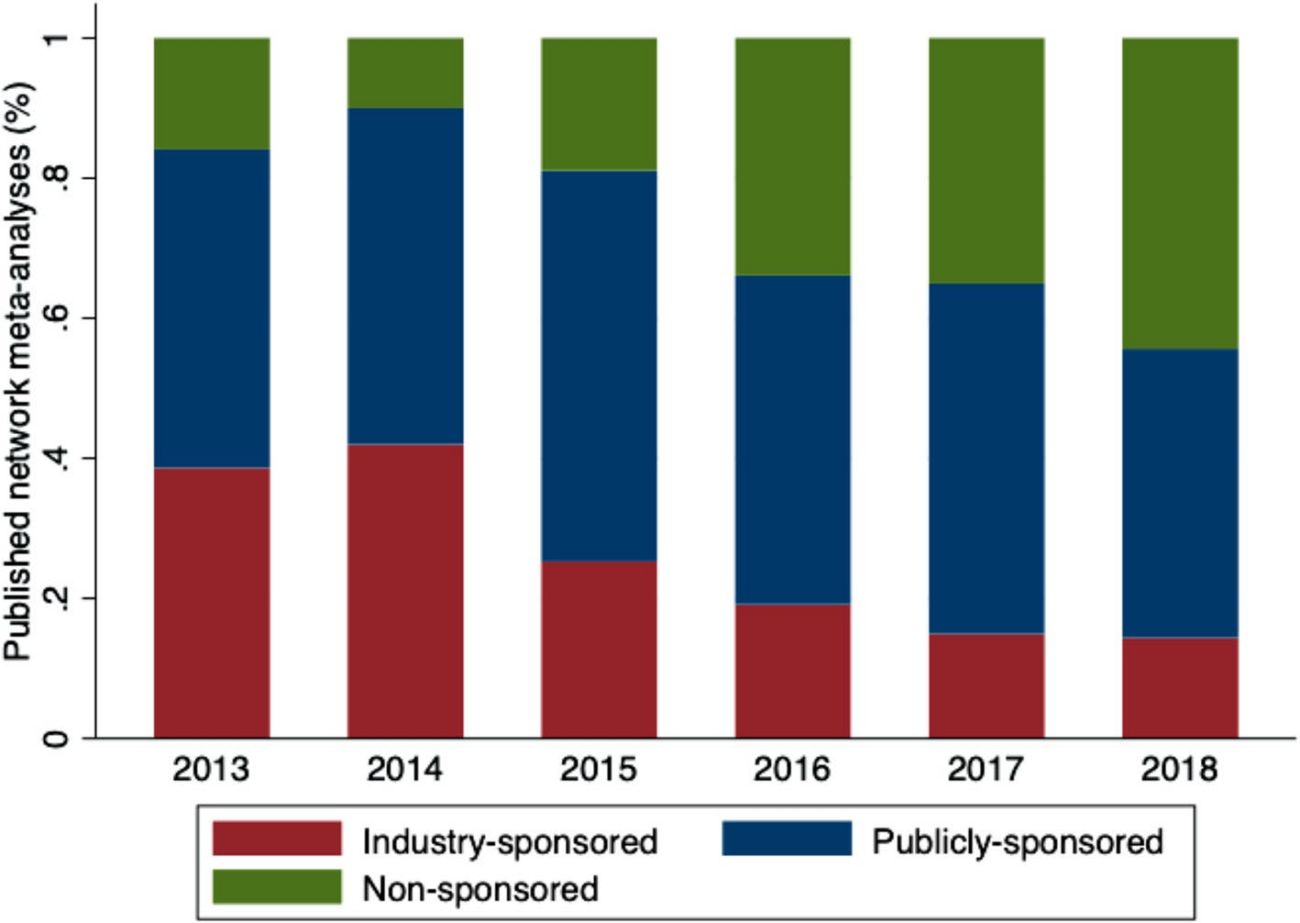
Stacked bar plot of published network meta-analyses over year of publication and by type of funding. Note: Industry- and mixed-sponsored studies were combined in one group

The number of private industry-sponsored NMAs decreased with year of publication (e.g., 39% in 2013 vs 14% in 2018), whereas non-sponsored NMAs increased (e.g., 16% in 2013 vs 44% in 2018) and the amount of publicly-sponsored NMAs remained unchanged (e.g., 45% in 2013 vs 44% in 2018; Fig. 1). NMAs were published in journals with a median impact factor 3.86 (IQR 2.72–5.90). Private industry-sponsored NMAs were published in journals with lower impact factors (median 3.00 [IQR 2.27–4.16]) compared to publicly- and non-sponsored NMAs (publicly-sponsored NMAs: median 4.39 [IQR 2.97– 7.09], non-sponsored NMAs: median 3.74 [IQR 2.69–5.24]; Appendix 4 (Additional file 1), on average.

### Author COI characteristics by funding type

#### Across all NMAs (*n* = 658)

The median number of authors in a NMA was seven (IQR 5–9). On average and across NMAs, 15% of the total authors included in a NMA were affiliated with the study funder or with any for-profit company. Similarly, 17% of authors declared any financial COIs in a NMA, on average. Overall, 136 (21%) NMAs included industry employees (Appendix 5 (Additional file 1)).

#### Industry-sponsored NMAs (*n* = 136)

The median number of authors in an industry-sponsored NMA was six (IQR 5–8). Most NMAs (108 [79%]) included industry employees. In 24 (18%) NMAs, the first author was affiliated with the funder, and they declared financial COIs with the funder in 103 (76%) NMAs (Appendix 5 (Additional file 1)). Similarly, the senior author was affiliated in 58 (43%) NMAs with the funder, and they declared any financial COI in 108 (79%) NMAs. Of the 24 (18%) first authors who were funder-affiliated, one (4%) author did not report their funder COI, and of the 58 (43%) senior authors who were funder-affiliated, seven (12%) authors did not report their funder COI.

The median number of authors affiliated with any funder was two (IQR 1–4), the number of authors affiliated with any for-profit company was five (IQR 2–6), and declared financial COIs was five (IQR 2–7). The most frequent type of financial funder-COI was employment for both first (19/24 [79%]) and senior (48/58 [83%]) funder-affiliated authors, and consultancy for both first (48/112 [43%]) and senior (35/78 [45%]) non-funder affiliated authors (Appendix 6 (Additional file 1)).

#### Publicly-sponsored NMAs (*n* = 314)

The median number of authors in a publicly-sponsored NMA was seven (IQR 6–9). In 58 (18%) NMAs the first author was affiliated with the public organization funder, and they declared any financial COI in 13 (4%) NMAs. Similarly, the senior author was affiliated in 57 (18%) NMAs with the public organization funder, and they declared financial funder COIs in 11 (3%) NMAs. Of the 58 first and 57 senior funder-affiliated authors none reported any COIs with the public funder.

### Reporting completeness of NMAs by funding type

**Fig. 2:**
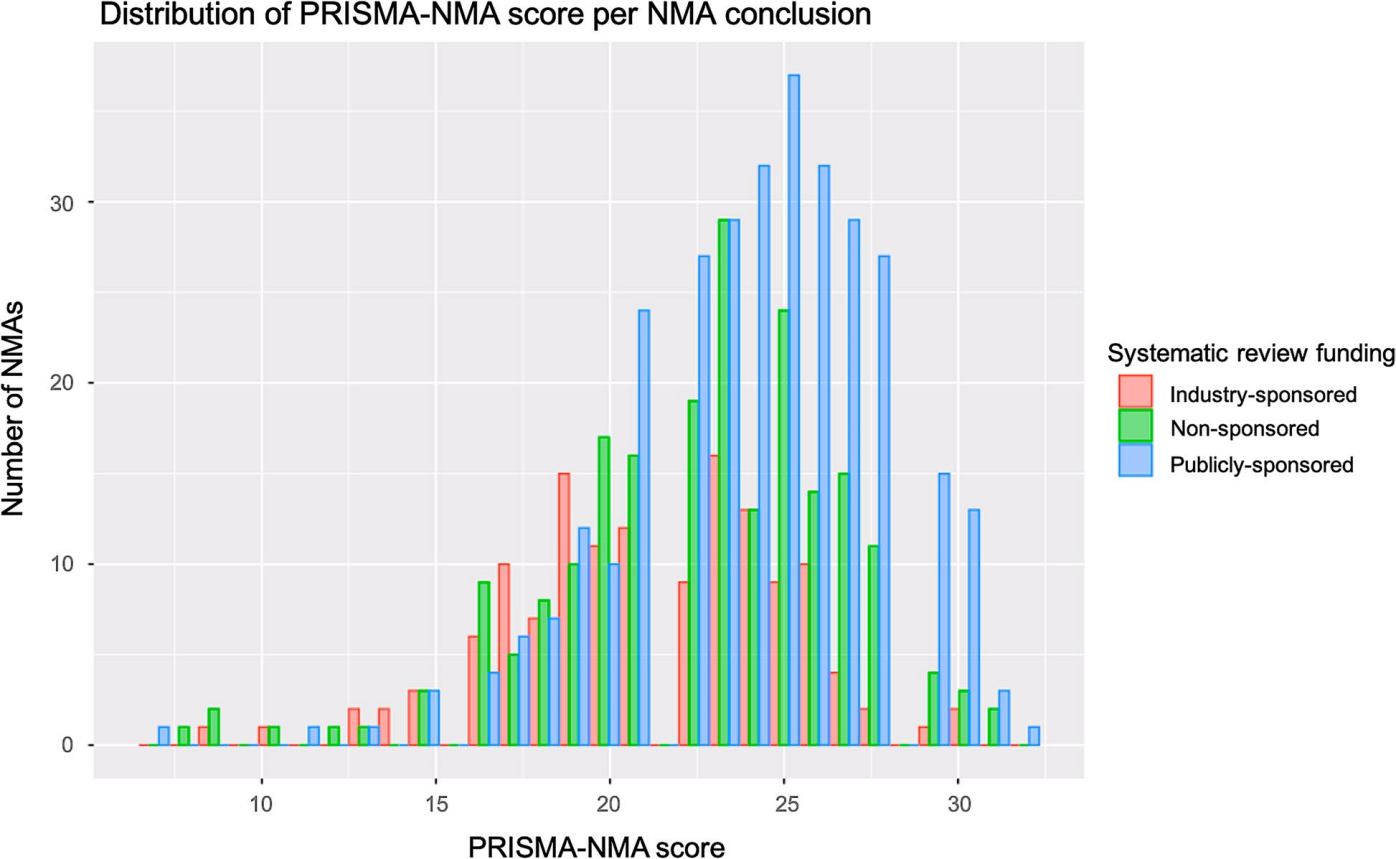
Histogram of PRISMA-NMA score by type of funding. Note: The PRISMA-NMA is a 32-item checklist

The median PRISMA-NMA score was 21 (IQR 19–24) in industry-sponsored NMAs, 23 (IQR 20–25) in non-sponsored NMAs, and 24.5 (IQR 22–27) in publicly-sponsored NMAs (total NMAs 23 [IQR 21–26], Fig. 2). Overall, reporting in NMAs of efficacy did not differ from NMAs of safety (median PRISMA-NMA score: efficacy 23 [IQR 21–26], safety 23 [IQR 20–26]). However, as expected, the median PRISMA-NMA scores differed by funding type in efficacy and safety NMAs (*efficacy*: industry-sponsored 21 [IQR 19–24], non-sponsored 23 IQR [20–25], publicly-sponsored 25 [IQR 22–27]; *safety*: industry-sponsored 20 [IQR 19–21], non-sponsored 23 IQR [20–25], publicly-sponsored 24 [IQR 21–26]). Of the total 658 NMAs, 243 (37%) did not report a within-study risk of bias assessment (i.e., present data on study risk of bias and outcome level assessment as suggested in the PRISMA-NMA), and this was mainly observed in 79/136 (58%) industry-sponsored NMAs (non-sponsored NMAs: 77/208 (37%); publicly-sponsored NMAs: 87/314 (28%); see Appendix 7 (Additional file 1)).

The PRISMA-NMA score did not differ by conclusion about the sponsored pharmacologic intervention in the primary outcome in industry-sponsored NMAs (Appendix 8 (Additional file 1)). However, the PRISMA-NMA score was higher in indeterminate overall NMA conclusions (median 24.5 [IQR 23–26]) compared to neutral and positive conclusions (median 21 [IQR 19–24]) in industry-sponsored NMAs (Appendix 9 (Additional file 1)).

### Treatment recommendation in 136 private industry-sponsored NMAs

Most (88 [65%]) industry-sponsored NMAs only recommended their own company’s intervention, 7 (5%) NMAs only recommended another company's drug, and 37 (27%) recommended both their own and another company's interventions, while 5 (3%) NMAs made no overall drug recommendations or suggested their drug is equivalent to their comparator(s) (Appendix 7 (Additional file 1)). Overall, 92% of the 136 NMAs recommended their company’s experimental intervention alone or in combination with another company’s drug. The primary outcome regarding the sponsored intervention was significantly positive in 112 NMAs (82%), and the overall NMA conclusion about the sponsored intervention was positive in 125 NMAs (92%).

Treatment effects were predominantly statistically significant irrespective of the year of publication; see absolute z-scores of the sponsored intervention compared to placebo/control in Appendix 10 (Additional file 1). Effect sizes along with their 95% CIs of the sponsored interventions are presented in Appendix 11 (Additional file 1).

### Matched comparison of industry-sponsored and non-industry sponsored NMAs

We were able to match 50 datasets (25 industry- with 25 non-industry sponsored datasets), using 36 unique NMAs; some NMAs reported or funded multiple pharmacologic interventions of interest (Appendix 12 (Additional file 1)). The private industry-sponsored NMAs had favourable conclusions more often than the non–industry-sponsored NMAs (in 100% vs 80% positive overall conclusions in the matched NMAs).

Of the 50 matched NMAs, we were able to compare 36 NMAs, resulting in 18 pairs of logORs or MDs. The remaining 14 NMAs, i.e., 7 paired datasets, did not report the same effect sizes and could not be compared. Eleven (61%) industry-sponsored NMAs were associated with larger efficacy effect sizes for the primary outcome, but these did not differ importantly compared with non-industry sponsored NMA effect sizes (Appendix 12, Appendix 13 (Additional file 1)). Of the 11 industry-sponsored NMAs, six (55%) assessed potential risks of bias in individual RCTs compared to the nine (82%) non-industry sponsored NMAs. The mean PRISMA-NMA score was lower for industry-sponsored NMAs compared to non-industry sponsored NMAs (22 vs 24).

## Discussion

Research focused on a specific drug which is funded by the manufacturer of the drug has found to be more prone to producing results favourable to the manufacturer’s intervention [15]. Our results showed that NMAs recommended their company’s experimental intervention alone or in combination with another company’s drug (92%), reported a statistically-significant effect estimate (82%) of their primary intervention’s outcome, and a positive conclusion (92%) across all outcomes. NMAs funded by drug companies were more likely to suggest favourable conclusions to the sponsor's product, and to estimate larger, but not significantly different, efficacy effect sizes for the primary outcome compared with NMAs funded by other sources. Possible reasons for more favourable results in industry-sponsored NMAs compared to non-industry sponsored NMAs may include implicit/unconscious biases that we are not able to measure, and the lack of assessing RCT risk of bias. Other reasons may include differences in the network geometries (including the number of nodes, studies, and participants) due to differences in the conduct of systematic review (e.g., literature searches, screening and data abstraction process), overall research question, including objectives of the review, PICO (i.e., patient, intervention, comparator, outcome) and eligibility criteria (e.g., population age, disease severity, performance status, and treatment doses), year of study publication, types of analyses and statistical modelling performed in the matched NMAs. Consideration of node making in a network is an important aspect, and relevant decisions, number of included interventions and nodes, along with rationale, should be presented at the protocol and registration stage. However, industry sponsorship did not appear to significantly influence effect sizes and their interpretation. Our conclusions are firmly supported by our data. In addition to reporting, future studies should explore whether quality and risk of bias is accounted in interpretation and conclusion of NMAs.

Reporting of NMAs of pharmacologic interventions can differ according to their source of funding (industry, public or non-sponsored NMAs). Of the three funding types, publicly-sponsored NMAs had the best reporting and published their findings in the highest impact-factor journals. Similarly, risk of bias assessments for the included RCTs were more frequently reported in publicly-sponsored NMAs than private industry-sponsored NMAs (72% vs 42%). However, the lead authors (first or senior) of the 314 publicly-sponsored NMAs, who were affiliated with the study’s public sponsor only rarely (first authors [4%]; senior author [3%]) declared their financial COIs with the public sponsor in the COI section. This assessment was only based on what was reported in the manuscript, and not on the author ICMJE forms submitted to the underlying journals. In the 136 industry-sponsored NMAs, senior authors were more frequently affiliated with the sponsor than first authors (43% vs 18%), but both declared their financial COIs with the industry sponsor in 3 out of 4 published NMAs.

This is the largest empirical study used to evaluate reporting in NMAs with different funding sources. We followed the JBI methods manual for conducting our scoping review [28] and the PRISMA extension for scoping reviews for reporting [29]. Overall, our results are aligned with previous studies comparing industry-funded and non-industry funded pairwise meta-analyses [14, 40]. Reporting completeness was found to be lower in industry funded meta-analyses and conclusions about the sponsored intervention was more favourable [14]. Our study adds to the literature by revealing the impact of industry funding on NMAs compared to pairwise meta-analyses, using a much larger sample of reviews (658 NMAs vs 39 meta-analyses [14] or 175 Cochrane reviews [40]) and not limiting by publication year or journal.

Selective reporting of outcomes in trial publications is common [41]. Outcomes in published manuscripts frequently do not match the pre-specified primary outcome in trial protocols, with positive outcomes more commonly presented in publications [42]. Industry-funded trials are significantly more likely to recommend the experimental intervention (odds ratio 5.3 95% CI [2.0–14.4]) [43]. When unpublished data from regulatory agencies were added to published meta-analyses, the summary efficacy estimates were lower in 46% of the reviews [44]. Similarly, serious adverse events were also inconsistently reported in published trials compared with trial registry data [45]. A qualitative study revealed that trial investigators were influenced by industry sponsors to selectively report outcomes [46]. Supplementing published data with trial registry results and the grey literature can help improve the quality of systematic reviews and NMAs [47].

Our study has a few limitations. First, we did not blind investigators to the information abstracted regarding the review authors, their affiliations, and their funding status. To avoid potential biases, we verified abstracted data with a second reviewer. Second, reporting assessment was conducted based on the information reported in the final publication, and we did not explore reporting in original protocols. Nevertheless, in our assessment, we considered all available supplementary files and appendices. Third, our database included NMAs published to July 2018, and we may have missed recent well-conducted and reported NMAs. However, our previous study showed that there are no major differences in completeness of reporting before and after the PRISMA-NMA publication, except for the items associated with NMA [27]. Also, given that industry-sponsored NMAs decreased over time, we expect that no major differences would be seen in our results if more recent NMAs would have been included in the database. In this study we assessed the largest number of reviews regarding recommendation rate of private industry-funded interventions. Fourth, in our matched NMAs we used repeated data when a NMA assessed multiple funded interventions. However, this was balanced in the two groups (five industry vs six non-industry repeated NMAs across the 50 matched NMAs), and hence we expect that this will not importantly impact our findings.

## Conclusions

Private industry sponsorship is a major source of funding for medical research, evidence synthesis and NMAs. However, decision-makers, such as patient partners, healthcare providers, policy-makers, and guideline-developers should be cautious when evaluating the results of industry-sponsored NMAs, since reporting completeness can be low. Also, COIs can influence the findings and conclusions of an NMA. Guidelines on how to report funding for the conducted NMA, and on how to report authors’ information, including their affiliations, funding, and financial COIs, are needed. For example, employees of the study sponsor should provide an interpretation of how their employment is relevant to or can impact the underlying research and interventions assessed along with their employment declaration. This would increase transparency and make findings better interpretable, irrespective NMA funding type.

## Supplementary Information

**Additional file 1:** **Supplementary Online Content (Appendices 1–13).** The document includes supplementary information (Appendices 1–13).

**Additional file 2.** PRISMA-ScR Checklist for Sponsorship in NMA. PRISMA-ScR checklist.

## Data Availability

The data that support the findings of this study are available from the corresponding author upon reasonable request.

## Abbreviations

RCTs: Randomised clinical trials
COIs: Conflict of interests
NMAs: Network meta-analyses
JBI: Joanna Briggs Institute
PRISMA: Preferred Items for Systematic Reviews and Meta-analysis
PRISMA-NMA: Preferred Reporting Items for Systematic Reviews and Meta-Analysis extension to NMA
IQR: Interquartile range
PROSPERO: International Prospective Register of Systematic Reviews
