# Supplementary material for: Does type of funding affect reporting in network meta-analysis? A scoping review of network meta-analyses
Source: Syst Rev. 2023 May 6;12:81. doi: 10.1186/s13643-023-02235-z (PMC10163730; doi:10.1186/s13643-023-02235-z)

Supplementary Online Content

[Appendix 1: Additional details on the methods 2](#_Toc130218330)

[Appendix 2: Flowchart for identification of eligible network meta-analyses (NMAs) using our previous NMA database. 8](#_Toc130218336)

[Appendix 3: Country of corresponding author in included NMAs by funding type 9](#_Toc130218337)

[Appendix 4: Distribution of journal impact factor, where network meta-analyses (NMAs) were published according to the systematic review funding type (SRfunding) 11](#_Toc130218338)

[Appendix 5: Author COI characteristics by funding type 12](#_Toc130218339)

[Appendix 6: Barplots depicting the frequency of conflict of interest (COI) type for the network meta-analysis (NMA) first and senior author according to their affiliation with the industry-sponsor. 13](#_Toc130218340)

[Appendix 7: Reporting characteristics by funding type 14](#_Toc130218341)

[Appendix 8: Boxplots of PRISMA-NMA score in industry-sponsored network meta-analyses (NMAs) by conclusion about the sponsored intervention in the primary outcome according to the calculated effect size. 15](#_Toc130218342)

[Appendix 9: Boxplots of PRISMA-NMA score in industry-sponsored network meta-analyses (NMAs) by overall conclusion about the sponsored intervention in the NMA 16](#_Toc130218343)

[Appendix 10: Boxplots of absolute z-score values by year of publication. The blue horizontal line represents the cut-off z-score value 1.96 17](#_Toc130218344)

[Appendix 11: Forest plots of effect sizes reported for the underlying industry-sponsored intervention 18](#_Toc130218345)

[Appendix 12: Characteristics of the matched industry- with non-industry-sponsored network meta-analyses (NMAs) comparing a pharmacologic intervention vs. placebo/control 21](#_Toc130218346)

[Appendix 13. Forest plots of effect sizes reported for the underlying industry-sponsored intervention. 24](#_Toc130218347)

# Appendix 1: Additional details on the methods

## **Search strategy**

We searched for NMAs of RCTs published in MEDLINE, EMBASE, PubMed, and Cochrane Database of Systematic Reviews up to July 2018.^25,28-30^

## **Inclusion criteria and study selection**

Similar to our previous publication, we included NMAs published between January 2013 and July 2018 to have an equal chronological timeframe before and after the PRISMA-NMA publication.^31^ We included networks of RCTs with at least four intervention nodes and a number of studies larger than the number of nodes compared (e.g., if four nodes were compared then five studies had to be included), where the authors had conducted an adjusted indirect comparison or NMA.^32^ We considered NMAs of direct and indirect evidence comparing pharmacologic interventions versus other pharmacologic interventions or placebo/control, since industry usually produces and funds pharmacologic interventions. A team member (AAV) contacted the corresponding authors of NMAs that had an unclear type of funding or did not report funding information in the publication or relevant PROSPERO registration for further clarification with up to two email reminders. If no clarification on funding was found or provided by the authors, the NMA was excluded. NMAs with non-pharmacologic interventions or comparisons also were excluded from the sample.

## **Data charting and data items**

We developed a predefined data abstraction form in an Excel spreadsheet (see data abstraction form below)^25^, and pilot tested it using five purposefully chosen NMAs. One review author (AAV, EW, CL, JCMM) performed data extraction and a second review author verified data (AAV, EW, CL, JCMM, IF). The data items abstracted from the included NMAs are described below.

We grouped NMAs according to their type of funding, irrespective of authors’ funding, as: industry-sponsored, publicly-sponsored, mixed-sponsored (i.e., includes studies funded by both public organizations and industry), and non-sponsored. We categorised NMAs according to the type of pharmacologic intervention comparisons as pharmacologic vs placebo or pharmacologic vs pharmacologic, depending on the presence of placebo or control.^30^ We classified the effect of the pharmacologic intervention of industry/mixed-sponsored NMAs as: significantly positive, non-significantly positive, neutral, significantly negative, and non-significantly negative. For example, a significantly positive effect would be chosen in the case of a statistically significant odds ratio in a positive outcome showing that higher values suggest a more efficacious/safe treatment compared to placebo/control. We also categorised NMAs according to their overall conclusion as: *positive* when the authors stated that there is evidence of efficacy (or safety); *negative* when the authors advised against the use of the intervention or it was not recommended; *neutral* when there was no evidence of efficacy (or safety) or the authors reported no opinion; and *indeterminate* when they stated that there is insufficient evidence or that more research is required.^33^

We captured whether the industry/mixed-sponsored NMAs recommended their own or another company’s pharmacologic intervention. We additionally matched industry/mixed- with publicly/non-sponsored NMAs addressing the same research question, as per the disease, primary outcome, and pharmacologic intervention compared to placebo/control.

## **Assessment of reporting completeness**

We assessed the reporting completeness of the NMAs using the PRISMA extension to NMAs (PRISMA-NMA)^32^ 32-item checklist, as described elsewhere^25^ by funding type.

## **Statistical analysis**

We assessed funding type by journal publication as per journal impact factor and by reporting as per PRISMA-NMA score. Journal impact factor was retrieved by Web of Science (year 2019), and when this was not available, we obtained impact factor from the relevant journal’s website. If an impact factor was not available for year 2019, it was retrieved from the previous in order available year. We evaluated the distribution of absolute z-score values of the pharmacologic intervention effect divided by its standard error reported for the sponsored pharmacologic intervention vs placebo/control using boxplots.

For our analyses we used both Stata/MP 14.0 (StataCorp LLC 2015 College Station, TX: Stata Statistical Software: Release 14) and R version 3.6.2 (R Development Core Team 2019).^33^

The following data items were abstracted from the included NMAs:

- Study characteristics: first author’s name, publication year, journal name, journal’s impact factor as indicated in the Web of Science report (year 2019), and country and continent of corresponding author.
- Outcome characteristics: primary outcome of the funded pharmacologic intervention (henceforth called the primary outcome), description of the outcome, categorisation of the outcome as (i), efficacy or safety, (ii) subjective objective or semi-objective, and (iii) broader disease category.
- Participant characteristics: participant age restriction, number of participants in the network.
- Intervention characteristics: name of the pharmacologic intervention, pharmacologic interventions manufactured by the sponsor of the NMA, number of pharmacologic interventions in the network, conclusions about sponsor’s own pharmacologic intervention in both abstract and text (henceforth simply called the conclusion), and whether the conclusion was positive or negative, NMA recommended own company's pharmacologic intervention, or another company’s pharmacologic intervention
- Network characteristics: number of primary studies, number of nodes, type of effect size (e.g., odds ratio), effect size and 95% confidence interval (CI) for the pharmacologic intervention compared with placebo/control, direction of the effect (higher vs lower better). When multiple pharmacologic interventions were manufactured by the industry sponsor, we calculated the mean effect size and standard error across the reported effect sizes.
- Funding characteristics: Type of funding reported, role of the funders, name of the main study sponsor.
- Author characteristics: affiliation with the funding sponsor (industry or public), financial COI, and type of COI (e.g., consultant, employee, advisory, grant) of first and senior author, number of authors declaring financial COIs with the study sponsor, number of authors affiliated with any for-profit company, and total number of authors.

***Data abstraction form***

| **Data item** | **Notes/options** |
| --- | --- |
| Reviewer | Initials of the study abstractor |
| Verifier | Initials of the person verifying the data |
| Study | Study ID |
| Author | Last name of first author |
| Country of study | Country of corresponding author |
| Year of publication |  |
| Journal |  |
| Journal impact factor |  |
| Title | Manuscript title |
| Primary outcome | Choose Efficacy over Safety for which a NMA/indirect comparison was conducted  If not reported, choose outcome reported in the title/abstract, otherwise use the first outcome reported in the text |
| Description of the outcome(s) |  |
| Efficacy or safety | Was the primary outcome efficacy or safety? |
| Outcome type | Objective  Semi-objective  Subjective  * We used the same categorization as in Turner et al: <https://pubmed.ncbi.nlm.nih.gov/22461129/> |
| Disease name |  |
| Disease category | Area of study  e.g. Cardiology, Respirology, Dermatology, Obstetrics and gynaecology |
| Age restriction | Yes  No |
| Age comment | Indicate what was the age restriction if present |
| NMA funding type | Non-sponsored  Industry sponsored  Publicly sponsored  Mixed funding |
| Comparison | Pharmacological vs. pharmacological  Pharmacological vs. placebo  * We used the same categorization as in Turner et al: <https://pubmed.ncbi.nlm.nih.gov/22461129/>  And Nikolakopoulou et al:  <https://pubmed.ncbi.nlm.nih.gov/24466222/> |
| Control | Name of the active comparator or placebo. |
| Number of interventions in NMA (primary outcome) | Number of interventions irrespective of nodes (plus placebo). |
| Number of nodes in NMA (primary outcome) | Number of nodes in the network  * Include the placebo/control node in counting |
| Number of trials in NMA (primary outcome) | Count the number of trials in the primary outcome |
| Number of participants in NMA (primary outcome) | Calculate total number of participants if a summary table is available in paper |
| Number of treatments sponsored |  |
| Name of the sponsored treatment |  |
| Link on the web found about the drug information |  |
| Effect size | If multiple treatments were manufactured by sponsor, then use the average of all effect sizes.  Consider only comparisons treatment vs. placebo/control |
| Type of effect size | e.g., MD, SMD, RD, OR, RR |
| Direction: higher vs lower better | If negative shows an efficacious/safe treatment then choose lower.  If positive shows an efficacious/safe treatment then choose higher. |
| Results about sponsored intervention (if applicable) | Significantly positive  Non-significantly positive  Neutral  Significantly negative  Non-significantly negative  * Focus on the primary outcome only |
| NMA conclusion positive or negative | Positive (i.e., authors stated that there is evidence of effectiveness)  Negative (i.e., authors advised against the use of the intervention or it was not recommended)  Neutral (no evidence of effectiveness or they reported no opinion)  Indeterminate (i.e., stated that there is insufficient evidence or that more research is required)  Non-comparative  * Look at the paper’s abstract conclusion or discussion section to determine. We used the same categorization as in Tricco et al: <https://pubmed.ncbi.nlm.nih.gov/19128940/> |
| Interventions in NMA were manufactured by the industry sponsor | Yes  No |
| NMA recommended own company's drug | Yes  No |
| NMA recommended another company's drug | Yes  No |
| SR Source of funding reported | Yes  No |
| Role of funders for SR reported | Yes  No |
| Funding from manufacturers reported | Yes  No |
| Authors are content experts with conflicts of interest reported | Yes  No |
| Main study sponsor | If multiple manufacturer sponsors, then report all of them. |
| Comments on funding | Typically, there will only be 1 parent company sponsoring a study, but sometimes more than one company sponsors. |
| Authors include industry employees | Yes  No |
| Risk of bias assessment | Yes  No |
| PRISMA score | Use 32-point scale as in Veroniki et al:  <https://pubmed.ncbi.nlm.nih.gov/34507621/> |
| Is the first author affiliated with the manufacturer or the funding company? | Yes  No |
| Is the last author affiliated with the manufacturer or the funding company? | Yes  No |
| Has first author declared financial COIs with the study sponsor (e.g., speaker, grants, advisor, board member for the company?) | Yes  No |
| Type of financial COIs for first author | If previous question is answered with a YES, then specify. For example:   - industry board member - industry consultant - provided expert testimony - reported equity holdings - received gifts - holds patents - received payment for manuscript preparation - received research funding from industry - received royalties - received speaker fees/payment for the development of presentations from industry - received travel reimbursement from industry - Unspecified Financial COIs |
| Has last author declared financial COIs with the study sponsor (e.g., speaker, grants, advisor, board member for the company?) | Yes  No |
| Type of financial COIs for last author | If previous question is answered with a YES, then specify. For example:   - industry board member - industry consultant - provided expert testimony - reported equity holdings - received gifts - holds patents - received payment for manuscript preparation - received research funding from industry - received royalties - received speaker fees/payment for the development of presentations from industry - received travel reimbursement from industry - Unspecified Financial COIs |
| Number of authors affiliated with the study sponsor (employment)? |  |
| Number of authors affiliated with *any* for-profit company (employment)? |  |
| Number of authors that declared financial COIs with the study sponsor (e.g., speaker, grants, advisor, board member for the company?) |  |
| Total number of authors in the NMA |  |

# Appendix 2: Flowchart for identification of eligible network meta-analyses (NMAs) using our previous NMA database.


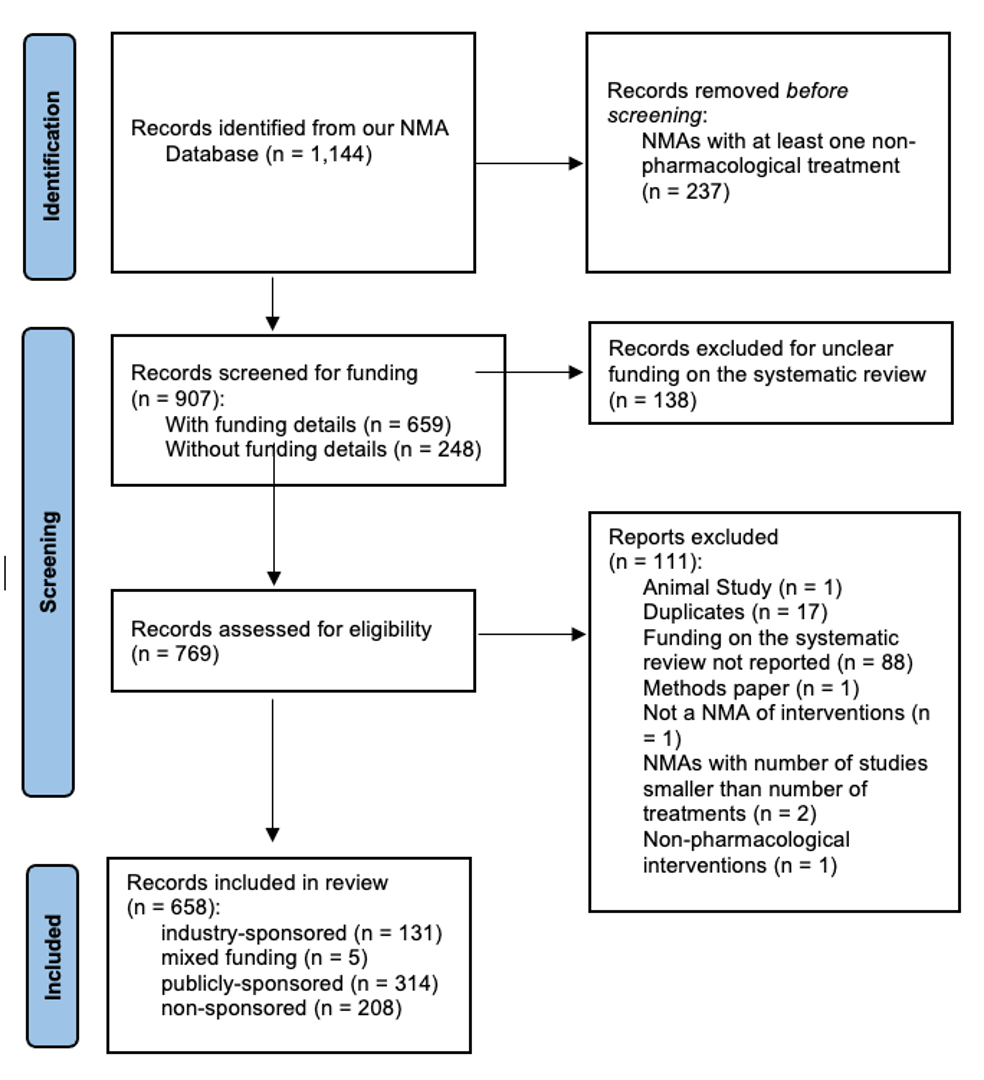


# Appendix 3: Country of corresponding author in included NMAs by funding type

|  | **Industry-sponsored (N=136)** | **Non-sponsored (N=208)** | **Publicly-sponsored (N=314)** | **Overall (N=658)** |
| --- | --- | --- | --- | --- |
| **Country of corresponding author** |  |  |  |  |
| Australia | 1 (1%) | 3 (1%) | 2 (1%) | 6 (1%) |
| Belgium | 2 (1%) | 0 (0%) | 0 (0%) | 2 (0%) |
| Canada | 13 (10%) | 12 (7%) | 20 (6%) | 45 (7%) |
| China | 4 (3%) | 63 (31%) | 131 (41%) | 198 (30%) |
| Denmark | 1 (1%) | 0 (0%) | 1 (0%) | 2 (0%) |
| France | 6 (4%) | 3 (1%) | 8 (3%) | 17 (3%) |
| Germany | 2 (1%) | 4 (2%) | 6 (2%) | 12 (2%) |
| Greece | 1 (1%) | 2 (1%) | 2 (1%) | 5 (1%) |
| Hungary | 2 (1%) | 0 (0%) | 0 (0%) | 2 (0%) |
| Iran | 1 (1%) | 0 (0%) | 1 (0%) | 2 (0%) |
| Ireland | 1 (1%) | 0 (0%) | 0 (0%) | 1 (0%) |
| Italy | 4 (3%) | 20 (11%) | 12 (4%) | 36 (5%) |
| Japan | 5 (4%) | 4 (2%) | 3 (1%) | 12 (2%) |
| Netherlands | 4 (3%) | 1 (0%) | 1 (0%) | 6 (1%) |
| Poland | 1 (1%) | 3 (1%) | 0 (0%) | 4 (1%) |
| South Africa | 1 (1%) | 0 (0%) | 0 (0%) | 1 (0%) |
| Switzerland | 2 (1%) | 1 (0%) | 3 (1%) | 6 (1%) |
| Taiwan | 1 (1%) | 1 (0%) | 8 (3%) | 10 (2%) |
| United Kingdom | 45 (33%) | 15 (8%) | 46 (15%) | 106 (16%) |
| United States of America | 39 (28%) | 32 (16%) | 42 (13%) | 113 (17%) |
| Argentina | 0 (0%) | 1 (0%) | 0 (0%) | 1 (0%) |
| Bahrain | 0 (0%) | 4 (2%) | 0 (0%) | 4 (1%) |
| Brazil | 0 (0%) | 5 (3%) | 6 (2%) | 11 (2%) |
| Colombia | 0 (0%) | 1 (0%) | 0 (0%) | 1 (0%) |
| Egypt | 0 (0%) | 2 (1%) | 0 (0%) | 2 (0%) |
| Fiji | 0 (0%) | 3 (1%) | 0 (0%) | 3 (0%) |
| Ghana | 0 (0%) | 1 (0%) | 0 (0%) | 1 (0%) |
| India | 0 (0%) | 2 (1%) | 0 (0%) | 2 (0%) |
| Israel | 0 (0%) | 1 (0%) | 0 (0%) | 1 (0%) |
| Lebanon | 0 (0%) | 1 (0%) | 0 (0%) | 1 (0%) |
| Malaysia | 0 (0%) | 5 (3%) | 0 (0%) | 5 (1%) |
| Norway | 0 (0%) | 3 (1%) | 1 (0%) | 4 (1%) |
| Portugal | 0 (0%) | 1 (0%) | 0 (0%) | 1 (0%) |
| Singapore | 0 (0%) | 4 (2%) | 0 (0%) | 4 (1%) |
| South Korea | 0 (0%) | 5 (3%) | 18 (6%) | 23 (4%) |
| Spain | 0 (0%) | 3 (1%) | 1 (0%) | 4 (1%) |
| Turkey | 0 (0%) | 2 (1%) | 0 (0%) | 2 (0%) |
| New Zealand | 0 (0%) | 0 (0%) | 2 (1%) | 2 (0%) |

# Appendix 4: Distribution of journal impact factor, where network meta-analyses (NMAs) were published according to the systematic review funding type (SRfunding)


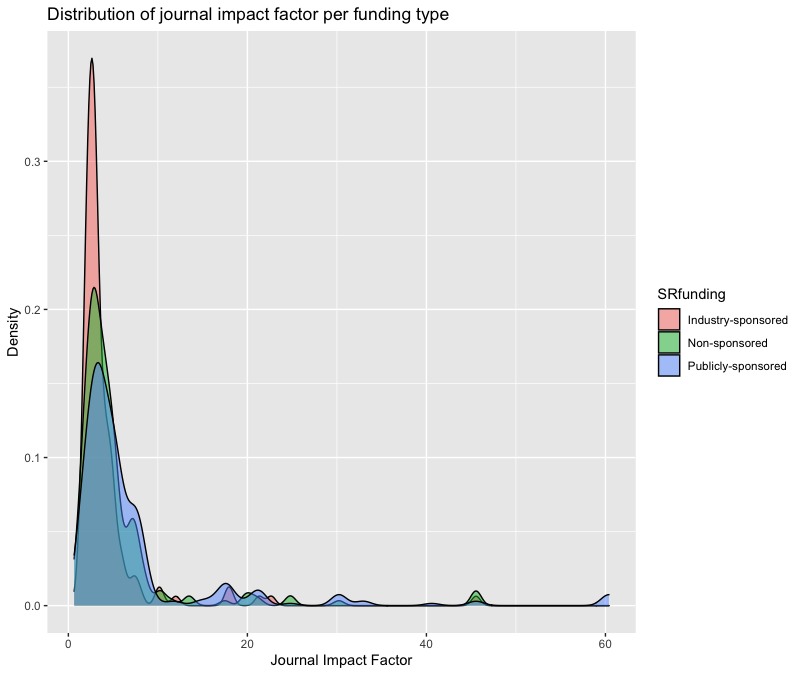


# Appendix 5: Author COI characteristics by funding type

|  | **Industry-sponsored (N=136)** | **Non-sponsored (N=208)** | **Publicly-sponsored (N=314)** | **Overall (N=658)** |
| --- | --- | --- | --- | --- |
| **Authors include industry employees** |  |  |  |  |
| No | 28 (21%) | 204 (98%) | 290 (92%) | 522 (79%) |
| Yes | 108 (79%) | 4 (2%) | 24 (8%) | 136 (21%) |
| **Is the first author affiliated with the manufacturer or the funding company?** |  |  |  |  |
| No | 112 (82%) | 208 (100%) | 256 (82%) | 576 (87%) |
| Yes | 24 (18%) | NA | 58 (18%) | 82 (13%) |
| **Has the first author declared financial COIs with the study sponsor?** |  |  |  |  |
| No | 33 (24%) | 208 (100%) | 301 (96%) | 542 (82%) |
| Yes | 103 (76%) | NA | 13 (4%) | 116 (18%) |
| **Is the senior author affiliated with the manufacturer or the funding company?** |  |  |  |  |
| No | 78 (57%) | 208 (100%) | 257 (82%) | 543 (82%) |
| Yes | 58 (43%) | NA | 57 (18%) | 115 (18%) |
| **Has the senior author declared financial COIs with the study sponsor?** |  |  |  |  |
| No | 28 (21%) | 205 (99%) | 303 (97%) | 536 (82%) |
| Yes | 108 (79%) | 3 (1%) | 11 (3%) | 122 (18%) |
| **Number of authors affiliated with the study sponsor?** |  |  |  |  |
| Median [IQR] | 2 [1–4] | NA | 0 [0–0] | 0 [0–1] |
| **Number of authors affiliated with any for-profit company?** |  |  |  |  |
| Median [IQR] | 5 [2–6] | NA | 0 [0–0] | 0 [0–0] |
| **Number of authors that declared financial COIs with the study sponsor?** |  |  |  |  |
| Median [IQR] | 5 [2–7] | NA | 0 [0–0] | 0 [0–0] |
| **Total Number of authors in the NMA** |  |  |  |  |
| Median [IQR] | 6 [5–8] | 6 [4–7] | 7 [6–9] | 7 [5–9] |

Abbreviations: COI, conflicts of interest; IQR, interquantile range; NA, not applicable; NMA, network meta-analysis

# Appendix 6: Barplots depicting the frequency of conflict of interest (COI) type for the network meta-analysis (NMA) first and senior author according to their affiliation with the industry-sponsor.

Note: Panel (a) corresponds to the first author not affiliated with the NMA industry-sponsor, panel (b) to the first author affiliated with the NMA industry-sponsor, panels (c) to the senior author not affiliated with the NMA industry-sponsor, and panel (d) to the senior author affiliated with the NMA industry-sponsor.


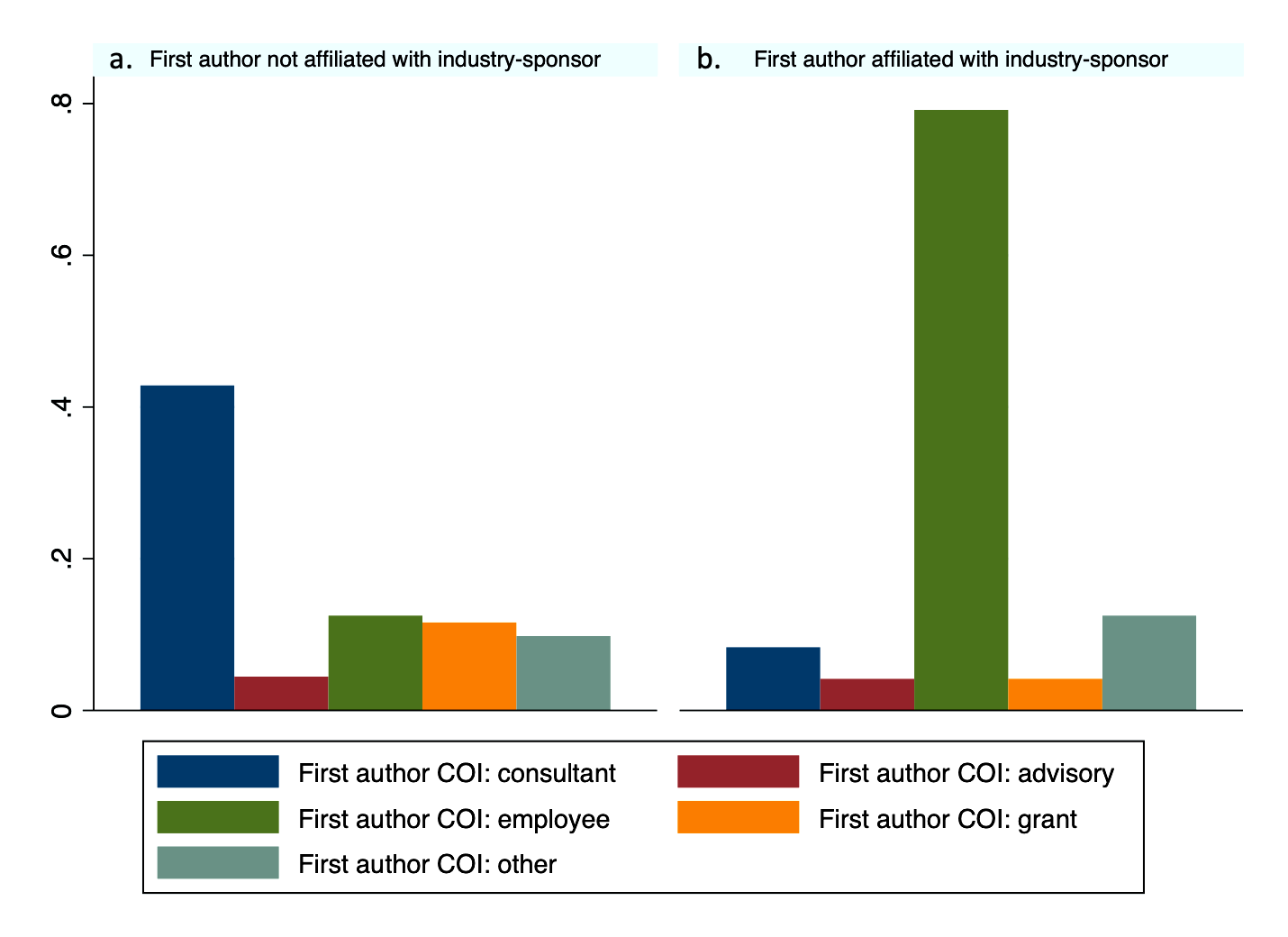


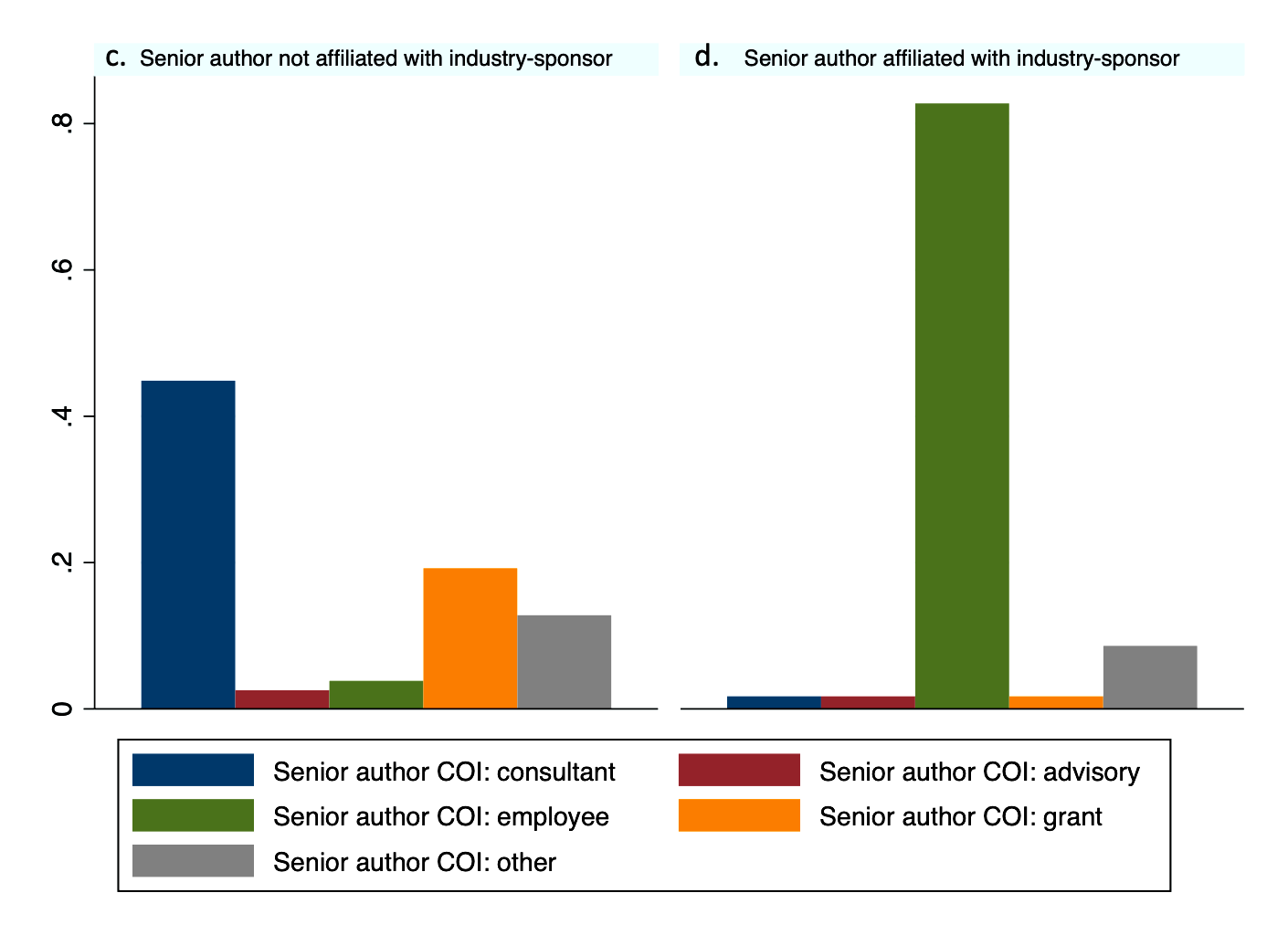


# Appendix 7: Reporting characteristics by funding type

|  | **Industry-sponsored (N=136)** | **Non-sponsored (N=208)** | **Publicly-sponsored (N=314)** | **Overall (N=658)** |
| --- | --- | --- | --- | --- |
| **PRISMA-NMA score (32-item checklist)** |  |  |  |  |
| Median [IQR] | 21  [19–24] | 23  [20–25] | 24.5  [22–27] | 23  [21–26] |
| **Risk of bias assessment of RCTs reported** |  |  |  |  |
| No | 79 (58%) | 77 (37%) | 87 (28%) | 243 (37%) |
| Yes | 57 (42%) | 131 (63%) | 227 (72%) | 415 (63%) |
| **NMA recommended own company's drug** |  |  |  |  |
| Not Applicable | 4 (3%)^ | 208 (100%) | 314 (100%) | 526 (80%) |
| No | 7 (5%) | NA | NA | 8 (1%) |
| Yes | 125 (92%)** | NA | NA | 124 (19%) |
| **NMA recommended another company's drug** |  |  |  |  |
| Not Applicable | 1 (0.74%) | 208 (100%) | 314 (100%) | 523 (79%) |
| No | 91 (67%) | NA | NA | 91 (14%) |
| Yes | 44 (32%) | NA | NA | 44 (7%) |
| **Conclusion about sponsored intervention*** |  |  |  |  |
| Not Applicable | 8 (6%)**^§^** | 208 (100%) | 314 (100%) | 530 (81%) |
| Non-significantly negative | 1 (1%) | NA | NA | 1 (0%) |
| Non-Significantly positive | 15 (11%) | NA | NA | 15 (2%) |
| Significantly positive | 112 (82%) | NA | NA | 112 (17%) |
| **Overall NMA conclusion about sponsored intervention** |  |  |  |  |
| Indeterminate | 2 (1%) | NA | NA | 2 (0%) |
| Not Applicable | 4 (3%)^ | 208 (100%)^§§^ | 314 (100%)^§§^ | 526 (80%) |
| Neutral | 5 (4%) | NA | NA | 5 (1%) |
| Positive | 125 (92%) | NA | NA | 125 (19%) |

* Characteristic as reported in the primary outcome of the systematic review with NMA

^§^4 NMAs did not include any interventions manufactured by the industry sponsor in the primary outcome, while another 4 NMAs did not report a numerical treatment effect estimate of the sponsored intervention in the publication

^ 4 NMAs did not include any interventions manufactured by the industry sponsor in the primary outcome

** 37/125 NMAs also recommended another company’s drug

^§§^ Non-industry NMAs were not assessed regarding a sponsored intervention, since they included multiple pharmaceutical products and conclusion would be irrelevant

Abbreviations: IQR, interquartile range; NA, not applicable; NMA, network meta-analysis

# Appendix 8: Boxplots of PRISMA-NMA score in industry-sponsored network meta-analyses (NMAs) by conclusion about the sponsored intervention in the primary outcome according to the calculated effect size.

Note: 11 industry-sponsored NMAs did not report a treatment effect along with a confidence interval, and were not included in this figure.


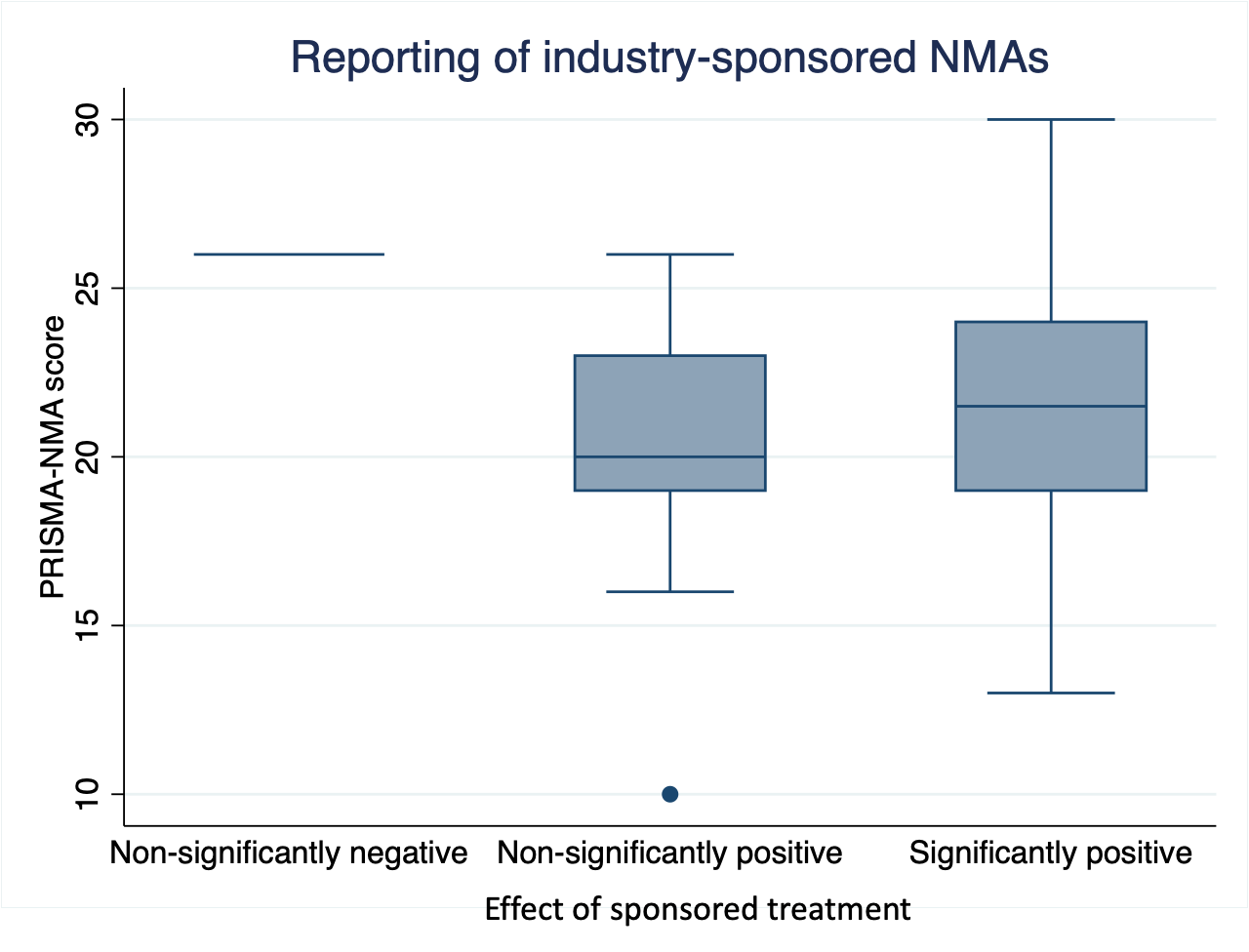


# Appendix 9: Boxplots of PRISMA-NMA score in industry-sponsored network meta-analyses (NMAs) by overall conclusion about the sponsored intervention in the NMA

Note: Four industry-sponsored NMAs did not include any interventions manufactured by the industry sponsor in the primary outcome (two were pharmacologic vs placebo and two were pharmacologic vs pharmacologic NMAs).


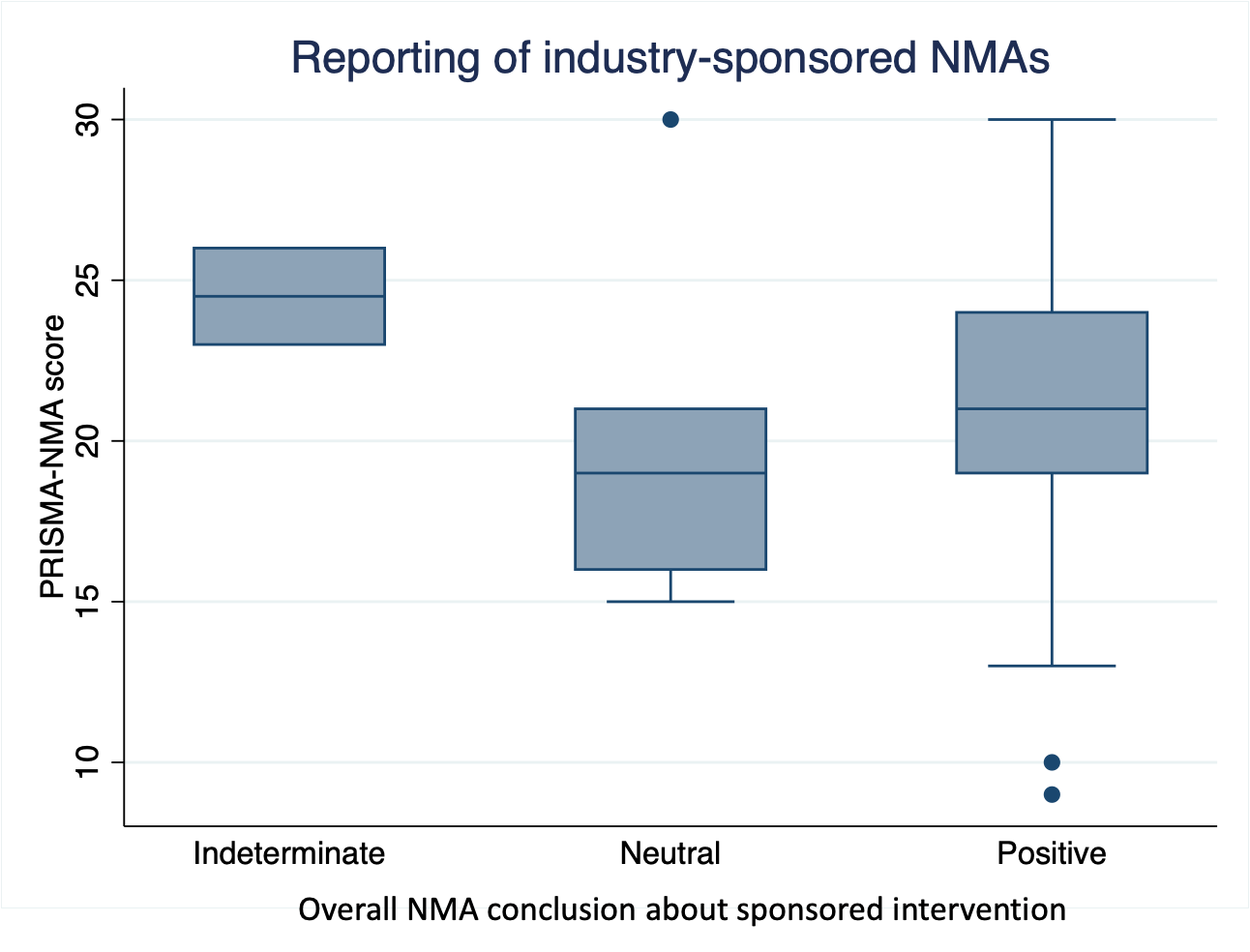


# Appendix 10: Boxplots of absolute z-score values by year of publication. The blue horizontal line represents the cut-off z-score value 1.96


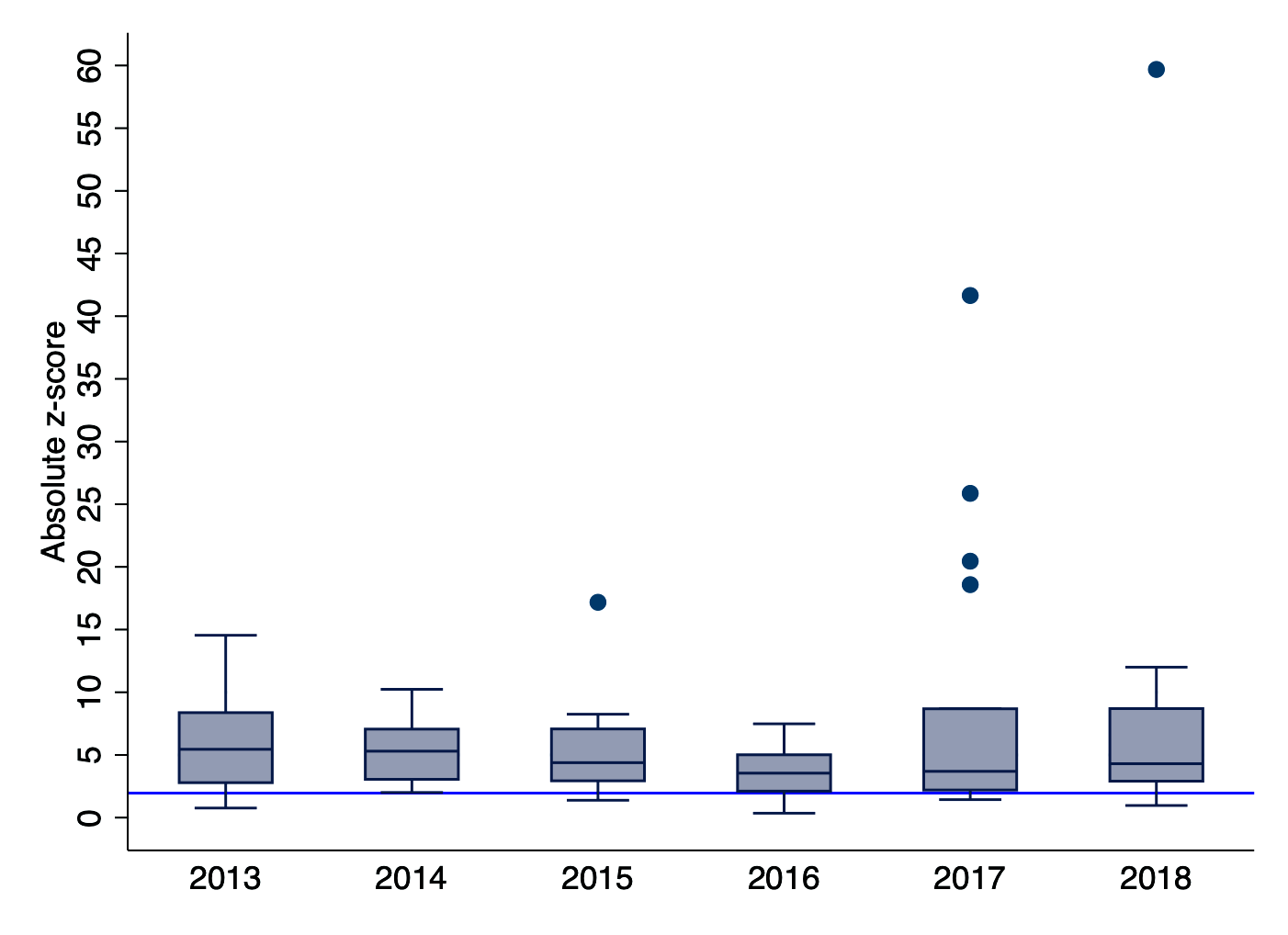


# Appendix 11: Forest plots of effect sizes reported for the underlying industry-sponsored intervention

Note: Panel corresponds to (a) log odds ratio effect sizes, panel (b) log risk ratio, hazard ratio and rate ratio effect sizes, (c) mean difference effect sizes, and (d) standardized mean difference effect sizes.

Notes:

1. In network meta-analyses (NMAs) with multiple effect sizes on the industry-sponsored intervention (e.g., multiple doses) or on several industry-sponsored interventions, results were combined using the average treatment effect and standard error.
2. In mean difference (MD) effect sizes, five NMAs were considered outliers with a MD>20, and were excluded from the forest plot.


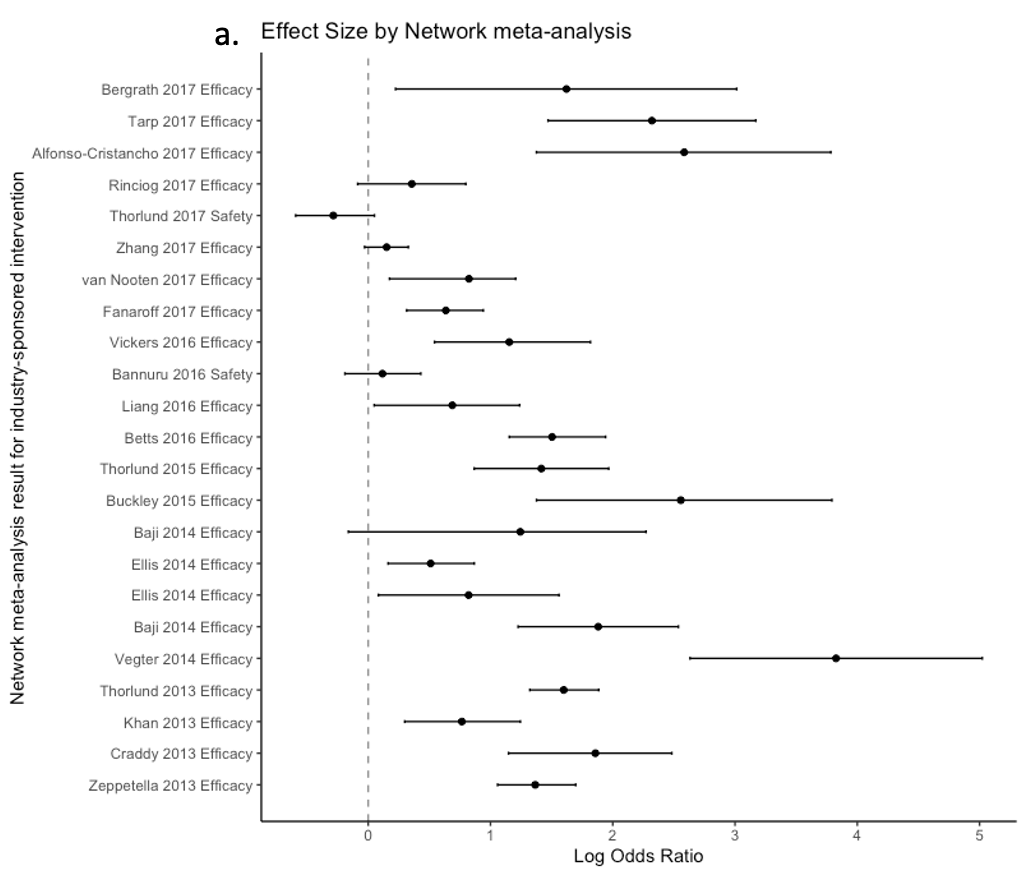


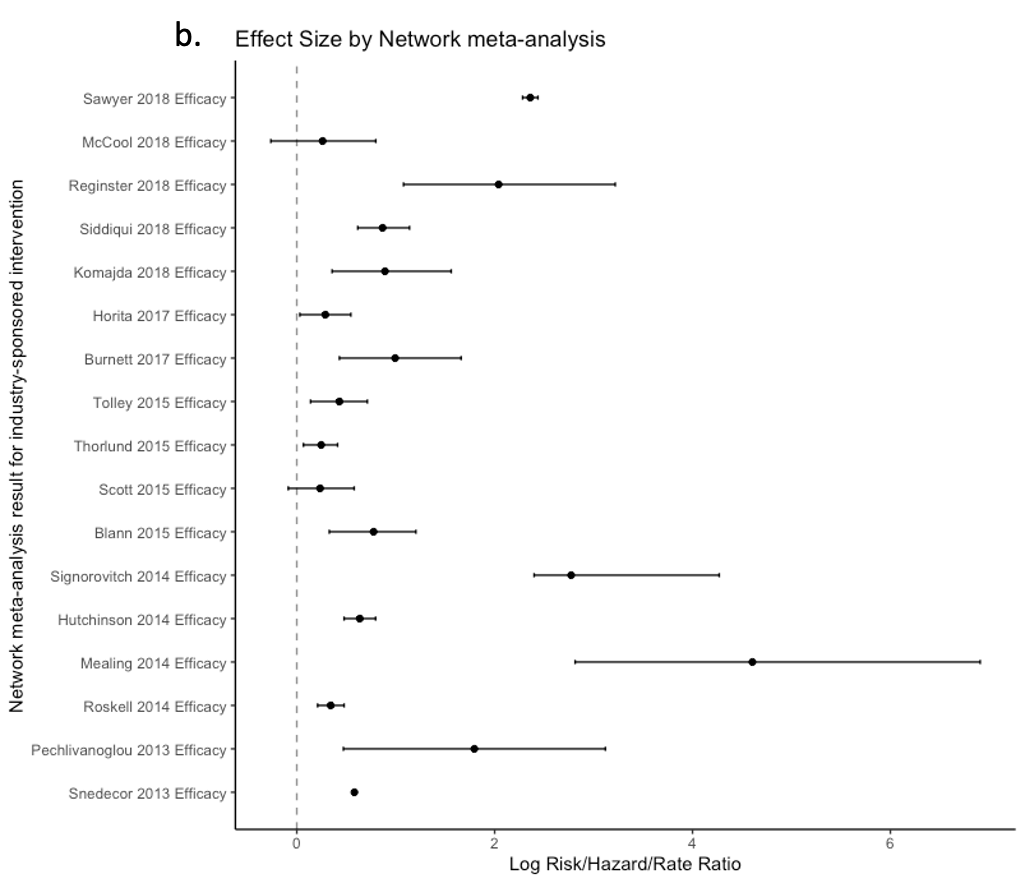


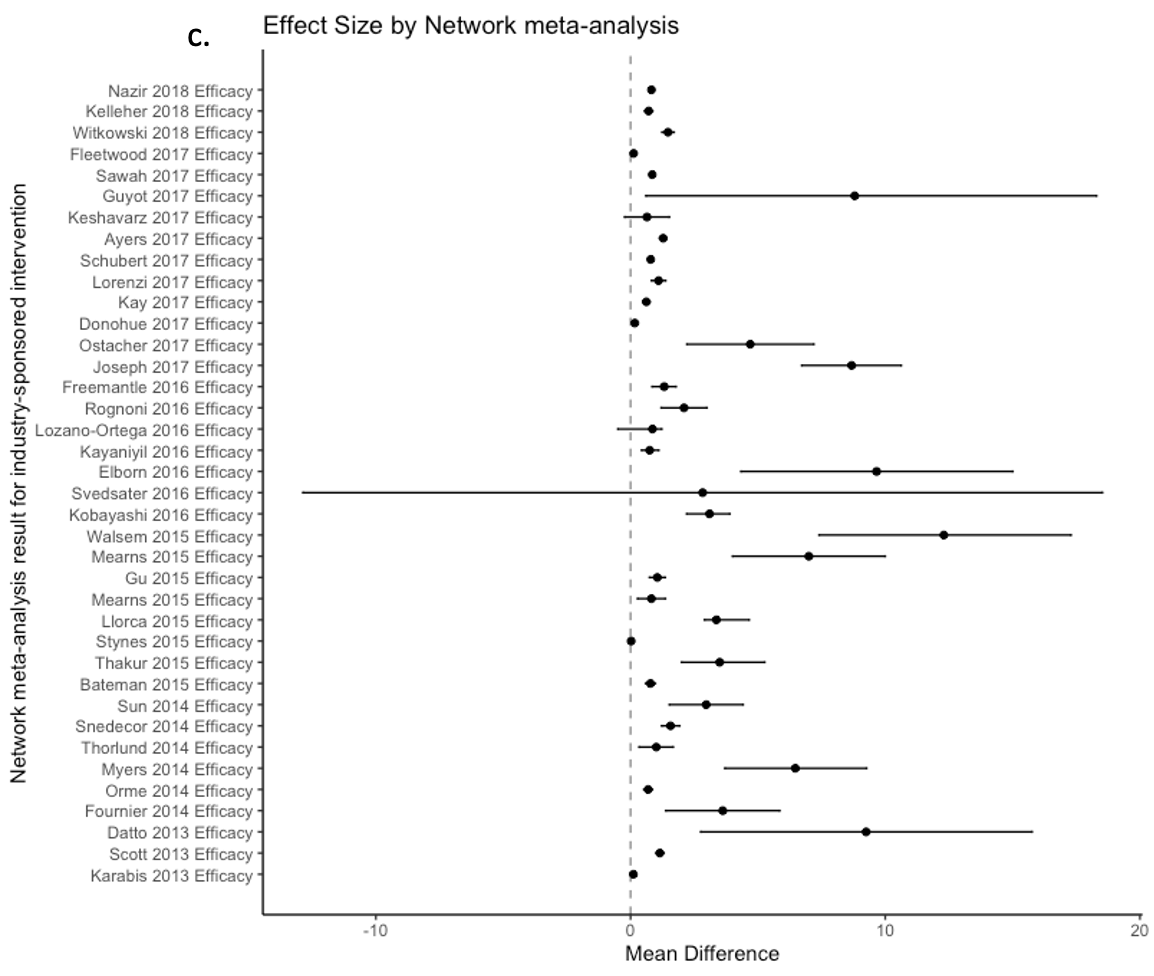


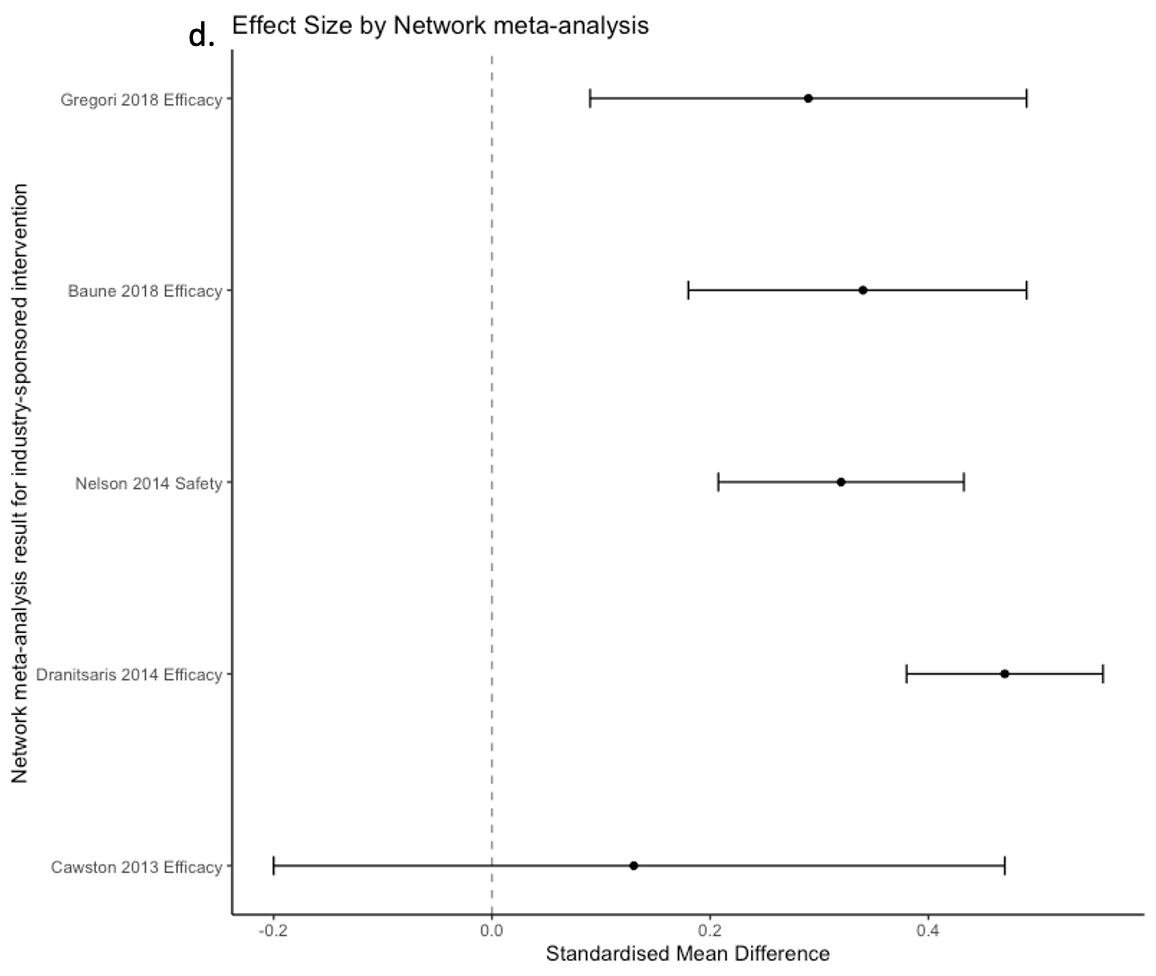


# Appendix 12: Characteristics of the matched industry- with non-industry-sponsored network meta-analyses (NMAs) comparing a pharmacologic intervention vs. placebo/control

| **Disease** | **Type of sponsor** | **Treatment** | **Study**  **(Author year)** | **Nodes in NMA*** | **Studies (participants) in NMA*** | **Effect size** | **Treatment effect (95%CI)*** | **Overall NMA conclusion** | **Difference in effect sizes**  **(95% CI)** |
| --- | --- | --- | --- | --- | --- | --- | --- | --- | --- |
| ADHD | Industry | lisdexamfetamine | Joseph 2017 | 5 | 20 (4667) | MD | 14.98  (12.80, 17.14) | Positive | 0.98  (-2.33, 4.29) |
| ADHD | Non-Industry  (Non-sponsored) | lisdexamfetamine | Li 2017 | 6 | 37 (NR) | MD | 14.00  (12.00, 17.00) | Positive |  |
| Ankylosing Spondylitis_2 | Industry | adalimumab | Betts 2016 | 7 | 15 (3094) | LOR | 1.50  (1.15, 1.94) | Positive | -0.11  (-0.74, 0.53) |
| Ankylosing Spondylitis_2 | Non-Industry  (Publicly-sponsored) | adalimumab | Liu 2016 | 5 | 16 (2574) | LOR | 1.61  (1.13, 2.12) | Positive |  |
| Ankylosing Spondylitis_4 | Industry | infliximab | Baji 2014 | 6 | 12 (2395) | LOR | 1.88  (1.23, 2.54) | Positive | 0.52  (-0.32, 1.36) |
| Ankylosing Spondylitis_4 | Non-Industry  (Publicly-sponsored) | infliximab | Liu 2016 | 5 | 16 (2574) | LOR | 1.36  (0.85, 1.91) | Positive |  |
| Diabetes_1 | Industry | dapagliflozin | Orme 2014 | 4 | 5 (1333) | MD | 0.69  (0.52, 0.86) | Positive | 0.03  (-0.16, 0.22) |
| Diabetes_1 | Non-Industry  (Publicly-sponsored) | dapagliflozin | Zaccardi 2016 | 10 | 38 (23997) | MD | 0.66  (0.58, 0.74) | Positive |  |
| Diabetes_2 | Industry | linagliptin | Mearns 2015 | 26 | 62 (32185) | MD | 0.64  (0.52, 0.77) | Positive | 0.03  (-0.13, 0.19) |
| Diabetes_2 | Non-Industry  (Publicly-sponsored) | linagliptin | Johnston 2017 | 11 | 18 (2942) | MD | 0.61  (0.51, 0.71) | Positive |  |
| Diabetes_3 | Industry | linagliptin | Mearns 2015 | 14 | 20 (6726) | MD | 0.60  (0.36, 0.84) | Positive | -0.01  (-0.27, 0.25) |
| Diabetes_3 | Non-Industry  (Publicly-sponsored) | linagliptin | Johnston 2017 | 11 | 18 (2942) | MD | 0.61  (0.51, 0.71) | Positive |  |
| Epilepsy | Industry | Perampanel | Khan 2013 | 5 | 12 (4303) | LOR | 0.77  (0.30, 1.24) | Positive | 0.06  (-0.78, 0.91) |
| Epilepsy | Non-Industry  (Publicly-sponsored) | Perampanel | Zhuo 2017 | 12 | 32 (7658) | LOR | 0.70  (0.01, 1.41) | Neutral |  |
| Fungal prophylaxis_3 | Industry | posaconazole | Pechlivanoglou 2013 | 9 | 25 (7062) | LRR | 2.12  (1.05, 3.22) | Positive | 0.46  (-0.78, 1.7) |
| Fungal prophylaxis_3 | Non-Industry  (Non-sponsored) | posaconazole | Lee 2018 | 14 | 54 (12832) | LRR | 1.66  (1.02, 2.21) | Positive |  |
| Fungal prophylaxis_4 | Industry | caspofungin | Pechlivanoglou 2013 | 9 | 25 (7062) | LRR | 1.47  (-0.13, 3.00) | Positive | 0.83  (-1.20, 2.87) |
| Fungal prophylaxis_4 | Non-Industry  (Non-sponsored) | caspofungin | Lee 2018 | 14 | 54 (12832) | LRR | 0.63  (-0.64, 1.97) | Neutral |  |
| Migraine | Industry | eletriptan | Thorlund 2013 | 8 | 74 (NR) | LOR | 1.60  (1.32, 1.89) | Positive | -0.70  (-1.46, 0.05) |
| Migraine | Non-Industry  (Non-sponsored) | eletriptan | Xu 2016 | 10 | NR (NR) | LOR | 2.30  (1.65, 3.04) | Positive |  |
| Multiple sclerosis_1 | Industry | BG-12 (dimethyl fumarate) | Hutchison 2014 | 8 | 27 (16530) | LRR | 0.64  (0.48, 0.8) | Positive | 0.39  (0.16, 0.62) |
| Multiple sclerosis_1 | Non-Industry  (Publicly-sponsored) | BG-12 (dimethyl fumarate) | Tramacere 2015 | 16 | 39 (25113) | LRR | 0.25  (0.08, 0.42) | Positive |  |
| Osteoporosis | Industry | Bazedoxifene | Ellis 2014 | 5 | 8 (16857) | LOR | 0.51  (0.16, 0.87) | Positive | -0.03  (-0.87, 0.80) |
| Osteoporosis | Non-Industry  (Publicly-sponsored) | Bazedoxifene | Liu 2018 | 5 | 7 (NR) | LOR | 0.54  (-0.20, 1.31) | Neutral |  |
| Prostate cancer | Industry | enzalutamide | McCool 2018 | 8 | 10 (6083) | LHR | 0.26  (0.13, 0.40) | Positive | -0.09  (-0.29, 0.11) |
| Prostate cancer | Non-Industry  (Publicly-sponsored) | enzalutamide | Wang 2018 | 17 | 26 (20314) | LHR | 0.35  (0.20, 0.50) | Positive |  |
| Psoriasis | Industry | brodalumab | Sawyer 2018 | 12 | 54 (17959) | LRR | 2.79  (2.61, 2.98) | Positive | -0.06  (-0.48, 0.36) |
| Psoriasis | Non-Industry  (Publicly-sponsored) | brodalumab | Loos 2018 | 9 | 34 (22892) | LRR | 2.85  (2.48, 3.23) | Positive |  |
| Pulmonary fibrosis | Industry | pirfenadone | Fleetwood 2017 | 5 | 9 (3581) | MD | 3.40  (1.90, 4.9) | Positive | 0.79  (-3.22, 4.80) |
| Pulmonary fibrosis | Non-Industry  (Publicly-sponsored) | pirfenadone | Loveman 2015 | 7 | 10 (891) | MD | 2.61  (-1.11, 6.33) | Neutral |  |
| Ulcerative colitis_1 | Industry | infliximab | Thorlund 2015 | 4 | 5 (NR) | LOR | 1.42  (0.87, 1.97) | Positive | 0.13  (-0.54, 0.80) |
| Ulcerative colitis_1 | Non-Industry  (Non-sponsored) | infliximab | Bonovas 2018 | 6 | 15(3130) | LOR | 1.29  (0.90, 1.67) | Positive |  |
| Ulcerative colitis_2 | Industry | golimumab | Thorlund 2015 | 4 | 5 (NR) | LOR | 0.93  (0.22, 1.59) | Positive | 0.18  (-0.58, 0.93) |
| Ulcerative colitis_2 | Non-Industry  (Non-sponsored) | golimumab | Bonovas 2018 | 6 | 15 (3130) | LOR | 0.76  (0.43, 1.08) | Positive |  |
| Ulcerative colitis_3 | Industry | Vedolizumab | Vickers 2016 | 5 | 7 (4052) | LOR | 1.15  (0.54, 1.82) | Positive | 0.00  (-0.89, 0.89) |
| Ulcerative colitis_3 | Non-Industry  (Non-sponsored) | Vedolizumab | Bonovas 2018 | 6 | 15 (3130) | LOR | 1.15  (0.54, 1.77) | Positive |  |
| Ankylosing Spondylitis_1 | Industry | adalimumab | Betts 2016 | 7 | 15 (3094) | LOR | 1.50  (1.15, 1.94) | Positive | Effect sizes cannot be compared |
| Ankylosing Spondylitis_1 | Non-Industry  (Publicly-sponsored) | adalimumab | Corbett 2016 | 6 | 26 (4596) | LRR | 0.82  (0.68, 0.96) | Positive |  |
| Ankylosing Spondylitis_3 | Industry | infliximab | Baji 2014 | 6 | 12 (2395) | LOR | 1.88  (1.23, 2.54) | Positive | Effect sizes cannot be compared |
| Ankylosing Spondylitis_3 | Non-Industry  (Publicly-sponsored) | infliximab | Corbett 2016 | 6 | 26 (4596) | LRR | 0.90  (0.55, 1.12) | Positive |  |
| Atrial fibrillation | Industry | edoxaban | Blann 2015 | 7 | 23 (71022) | LRR | 1.02  (0.58, 1.47) | Positive | Effect sizes cannot be compared |
| Atrial fibrillation | Non-Industry  (Publicly-sponsored) | edoxaban | Tawfik 2016 | 10 | 20 (96826) | LRtR | 0.99  (0.65, 1.35) | Positive |  |
| Fungal prophylaxis_1 | Industry | posaconazole | Pechlivanoglou 2013 | 9 | 25 (7062) | LRR | 2.12  (1.05, 3.22) | Positive | Effect sizes cannot be compared |
| Fungal prophylaxis_1 | Non-Industry  (Non-sponsored) | posaconazole | Zhao 2016 | 6 | 21 (5505) | LOR | 2.04  (1.2, 3.00) | Positive |  |
| Fungal prophylaxis_2 | Industry | posaconazole | Pechlivanoglou 2013 | 9 | 25 (7062) | LRR | 2.12  (1.05, 3.22) | Positive | Effect sizes cannot be compared |
| Fungal prophylaxis_2 | Non-Industry  (Publicly-sponsored) | posaconazole | Leonart 2017 | 11 | 25 (NR) | LOR | 1.75  (0.44, 3.01) | Positive |  |
| Heart failure | Industry | ARNI | Burnett 2017 | 11 | 57 (54993) | LHR | 0.99  (0.43, 1.66) | Positive | Effect sizes cannot be compared |
| Heart failure | Non-Industry  (Publicly-sponsored) | ARNI | Xie 2016 | 7 | 21 (69229) | LOR | 0.40  (0.15, 0.73) | Positive |  |
| Multiple sclerosis_2 | Industry | PEG IFN beta-1a | Tolley 2015 | 7 | 16 (8359) | LHR | 0.55  (0.12, 1.01) | Positive | Effect sizes cannot be compared |
| Multiple sclerosis_2 | Non-Industry  (Publicly-sponsored) | PEG IFN beta-1a | Tramacere 2015 | 16 | 39 (25113) | LRR | 0.12  (-0.12, 0.36) | Neutral |  |

*Information as reported in the primary outcome of the systematic review with NMA

Abbreviations: LOR, log odds ratio; LRR, log risk ratio; LRtR, log rate ratio; MD, mean difference; NMA, network meta-analysis

# Appendix 13. Forest plots of effect sizes reported for the underlying industry-sponsored intervention.

Note: Red font represents industry-sponsored NMAs correspond to, whereas blue font represents non-industry sponsored NMAs. Panel corresponds to (a) log odds ratio effect sizes, panel (b) mean difference effect sizes, and (c) mixed effect sizes, including log risk ratio, hazard ratio and rate ratio effect sizes.


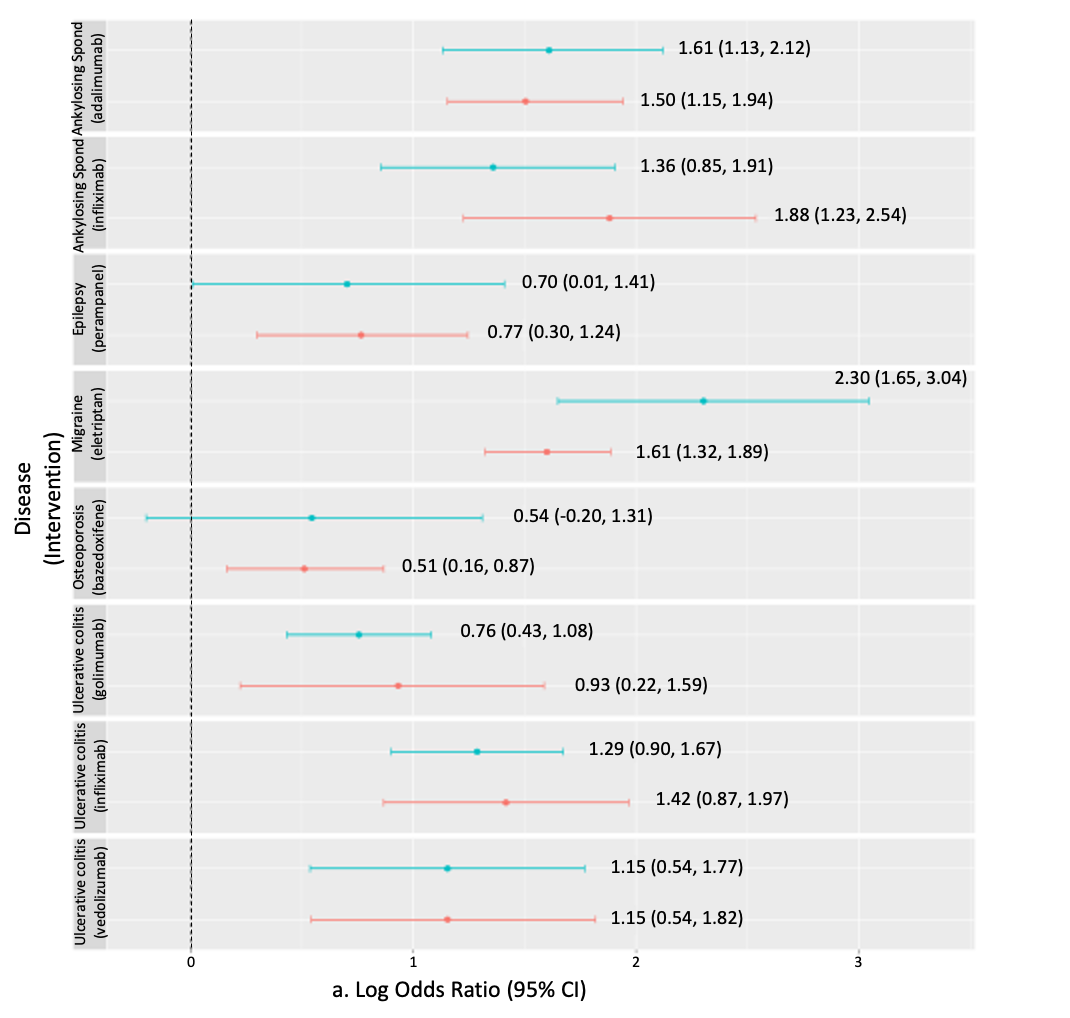


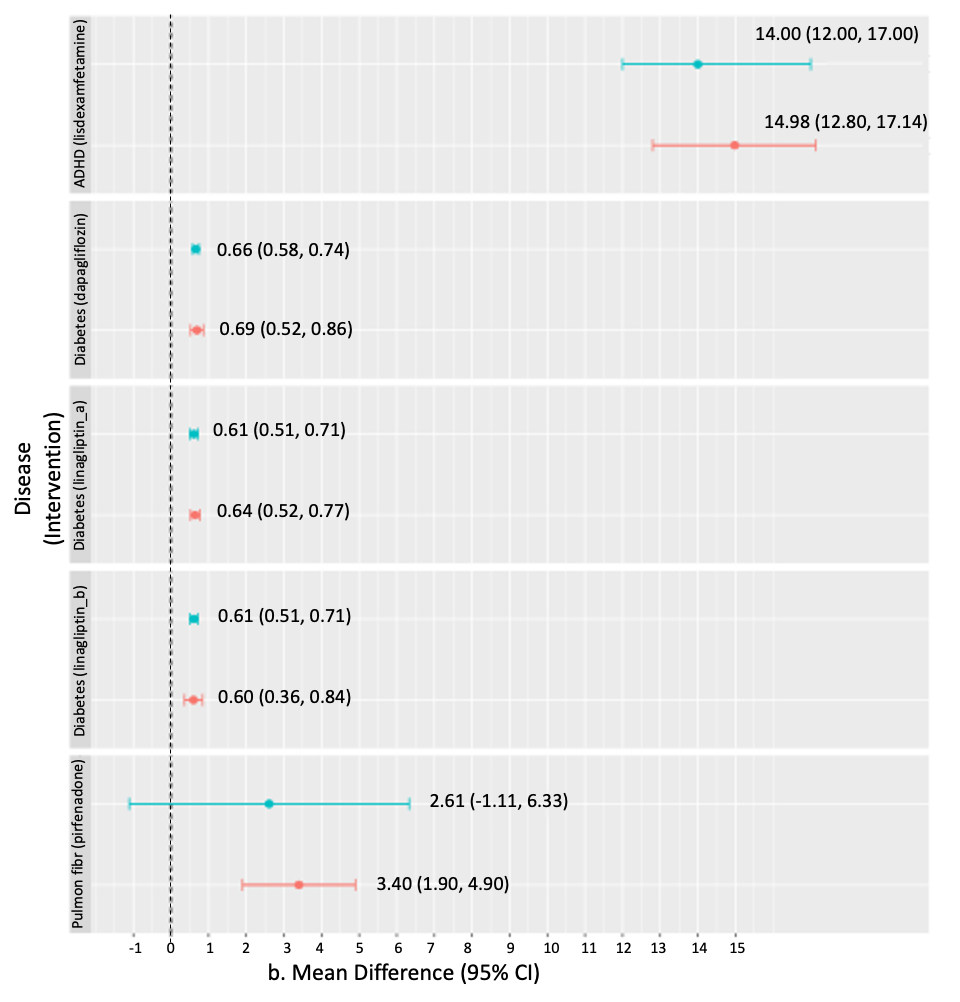


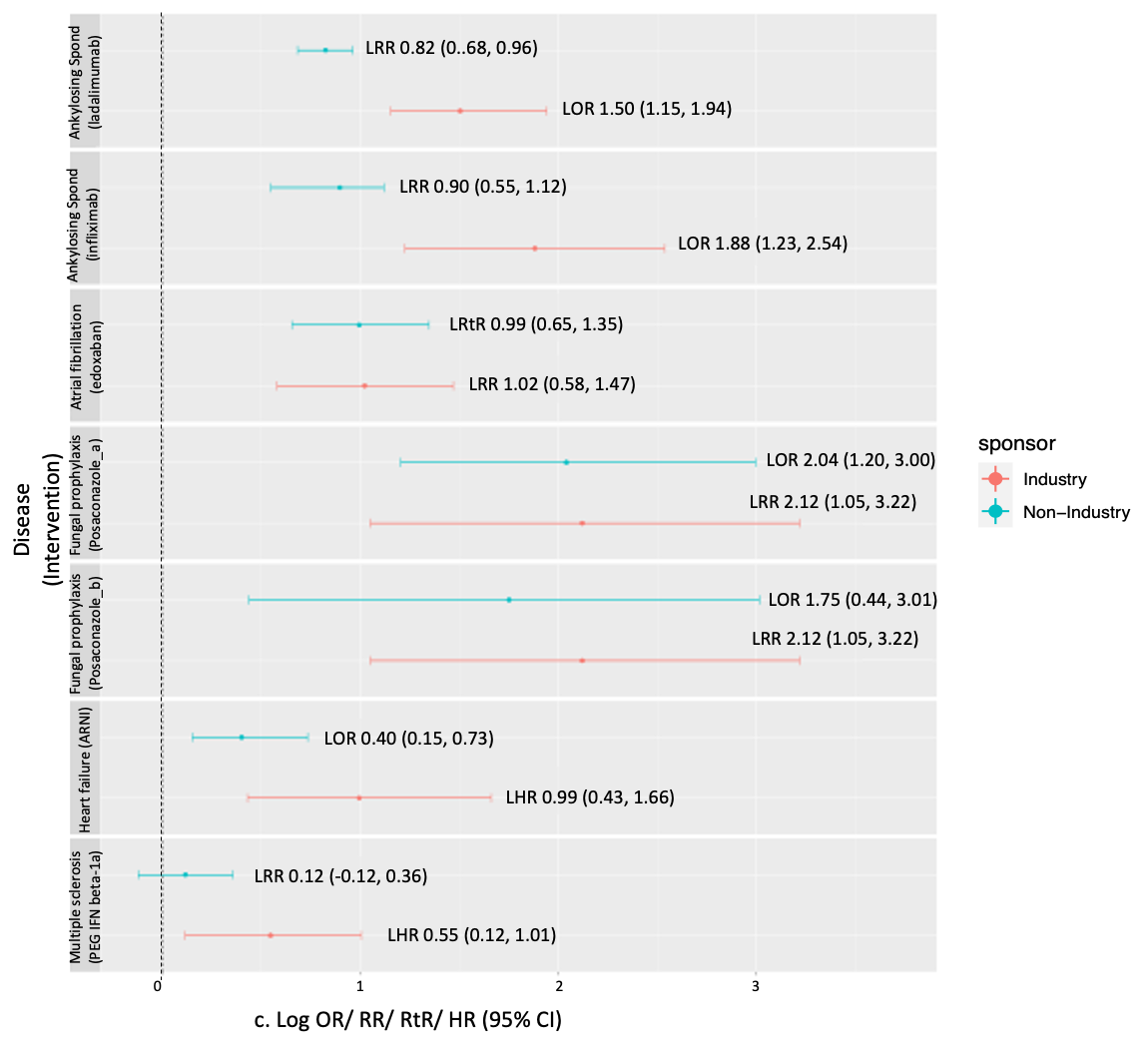

Supplement: Supplementary file 1 — Additional file 1: Supplementary Online Content (Appendices 1–13). The document includes supplementary information (Appendices 1–13). [file 13643_2023_2235_MOESM1_ESM.docx]
